# Transcutaneous Immunotherapy for RNAi: A Cascade‐Responsive Decomposable Nanocomplex Based on Polyphenol‐Mediated Framework Nucleic Acid in Psoriasis

**DOI:** 10.1002/advs.202303706

**Published:** 2023-10-05

**Authors:** Mei Zhang, Xin Qin, Yang Gao, Jiale Liang, Dexuan Xiao, Xiaolin Zhang, Mi Zhou, Yunfeng Lin

**Affiliations:** ^1^ State Key Laboratory of Oral Diseases, National Clinical Research Center for Oral Diseases, West China Hospital of Stomatology Sichuan University Chengdu 610041 P. R. China; ^2^ Department of Orthopedics, Orthopedic Research Institute, West China Hospital Sichuan University Chengdu 610041 P. R. China; ^3^ College of Biomedical Engineering Sichuan University Chengdu 610041 P. R. China

**Keywords:** DNA nanotechnology, lysosomal escape, psoriasis, siRNA delivery, tetrahedral frame nucleic acid

## Abstract

Skin is the first barrier against external threats, and skin immune dysfunction leads to multiple diseases. Psoriasis is an inflammatory, chronic, common, immune‐related skin disease that affects more than 125 million people worldwide. RNA interference (RNAi) therapy is superior to traditional therapies, but rapid degradation and poor cell uptake are the greatest obstacles to its clinical transformation. The transdermal delivery of siRNA and controllable assembly/disassembly of nanodrug delivery systems can maximize the therapeutic effect. Tetrahedral framework nucleic acid (tFNA) is undoubtedly the best carrier for the transdermal transport of genes due to its excellent noninvasive transdermal effect and editability. The authors combine acid‐responsive tannic acid (TA), RNase H‐responsive sequences, siRNA, and tFNA into a novel transdermal RNAi drug with controllable assembly and disassembly: STT. STT has heightened resistance to enzyme, serum, and lysosomal degradation, and its size is similar to that of tFNA, enabling easy transdermal transport. After transdermal administration, STT can specifically silence nuclear factor kappa‐B (NF‐κB) p65, thereby maintaining the stability of the skin's microenvironment and reshaping normal skin immune defense. This work demonstrates the advantages of STT in RNAi therapy and the potential for future treatment of skin‐related diseases.

## Graphical abstract

The cascade‐responsive decomposable nanocomplex (STT) consists of tetrahedral framework nucleic acid (tFNA), acid‐responsive tannic acid (TA), RNase H‐responsive sequences, and siRNA. STT is similar in size and structure to tFNA, which enables it to provide transdermal immunotherapy for psoriasis. STT can specifically silence nuclear factor kappa‐B p65, thereby maintaining the stability of the skin's microenvironment.

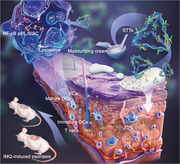

## 1 Introduction

Skin is a critical part of the host immune defense, serving as the first line of defense against external threats.^[^ ^1^ ^]^ Dysfunction of the dermal immune system and its role as a barrier are the basis of common chronic inflammatory disorders such as psoriasis and atopic dermatitis (AD).^[^ ^2^ ^]^ Psoriasis can cause severe pain, disfigurement and disability, and it affects approximately 2–3% of the world's population.^[^ ^3^ ^]^ Psoriasis is the result of the interplay between hyperproliferative keratinocytes (KCs) and immune cells.^[^ ^4^ ^]^ There is no drug that can completely cure psoriasis at present. Traditional local therapeutic and oral drugs, such as corticosteroids and methotrexate (MTX), have limited efficacy, serious hepatorenal toxicity and even teratogenicity.^[^ ^5^ ^]^ In some studies of biotherapy targeting the immune system, it was found that interleukin (IL)−17, tumor necrosis factor (TNF)‐α, and IL‐23 play more critical roles in the pathological mechanisms of psoriasis than other cytokines, and in particular, TNF‐α and IL‐17 exert a synergistic effect.^[^ ^6^ ^]^ Biological inhibitors of TNF‐α and IL‐23/IL17 have also become relatively effective first‐line interventions in recent years.^[^ ^3^ ^]^ Blocking TNF‐α and IL‐17 at the same time may more effective than blocking a single target. Nuclear factor kappa‐B (NF‐κB) is the direct transcriptional activator of TNF‐α and IL‐17A, and because it is the main regulator of the immune response, targeting should benefit a large group of patients.^[^ ^7^ ^]^ Therefore, we chose NF‐κB as the treatment target to block the inflammatory cycle of psoriasis to maximize patient benefits. However, the development of biological agents was limited by high cost and immunogenicity, and the limited injection delivery method introduces a risk of serious infections for the host.^[^ ^8^ ^]^

RNA interference (RNAi) therapy has become a relatively safe, accurate and personalized drug due to its highly specific combination with molecular targets.^[^ ^8^ ^]^ However, to date, only five siRNAs have been approved by the Food and Drug Administration (FDA).^[^ ^9^ ^]^ There are many difficulties in the development of siRNA, especially its rapid degradation, cycling, and clearance.^[^ ^10^ ^]^ Promising chemical modifications have been introduced to improve the stability of siRNA. However, some modifications also inhibit the entry of siRNA into the RNA‐induced silencing complex (RISC).^[^ ^11^ ^]^ Moreover, the effect of intracellular barriers on siRNA results in lysosomal degradation or limited uptake of most siRNAs.^[^ ^10a^ ^]^ In addition, rapid renal metabolism makes systemic infusion unsuitable, while local injection poses a risk of pain and infection.^[^ ^11^, ^12^ ^]^ Therefore, transdermal RNAi therapy has been launched, and this delivery method shows great advantages because it is painless and evades metabolic clearance, enzyme degradation and circulating clearance.^[^ ^12^, ^13^ ^]^ Moreover, skin is a highly immunogenic site containing many immune cells, such as KCs, dendritic cells (DCs), and T cells, which are the natural target of immunotherapy. However, the barrier function of skin and its dense structure pose a great challenge to the transdermal delivery of genes.^[^ ^12^ ^]^ Various physical and chemical methods have been developed for the transdermal administration of drugs, such as liposomes, peptides, microneedles, ion electroosmosis and electroporation, which are currently commonly used delivery methods. However, gene delivery is still affected by tight cell connections and cannot be spread. In addition, the problems of cell uptake and gene stability have not been solved.^[^ ^12^, ^14^ ^]^ Therefore, the development of intelligent nanocarriers that can be loaded/unloaded through transdermal drug delivery will minimize the drawbacks of RNAi therapy. The smart carrier must achieve the following: (1) stability of siRNA during delivery; (2) efficient transdermal delivery capability; (3) efficient cell uptake; and (4) successful release of siRNA drugs into the cytoplasm to perform their function.

Functional nucleic acid nanomaterials (FNANs), because of their sequence editability and high biocompatibility, have advantages in gene and drug delivery over other organic and inorganic carriers.^[^ ^14^, ^15^ ^]^ Genes and responsive structures can be successfully assembled on the FNAN structure through unique covalent binding or molecular recognition to form a whole. The structural integrity enhances the stability of the original single gene segment. The responsive structure allows the gene to be released in a programmed manner, which improves the utilization rate.^[^ ^14^, ^16^ ^]^ Specifically, the framework FNAN can easily cross the cell membrane barrier because of its unique spatial structure and has high cell uptake capacity.^[^ ^15b^ ^]^ Among framework FNANs, tetrahedral framework nucleic acid (tFNA) has attracted particular attention because of its remarkable cell uptake ability and tissue penetration ability.^[^ ^17^ ^]^ Moreover, tFNA has a relatively simple synthesis procedure and stable structure.^[^ ^18^ ^]^ Previous studies have shown that the size of drugs has a great impact on their transdermal ability. When tFNA side lengths of 37 bp and shorter were compared, a side length of 21 bp accumulated the largest amount in cells and skin tissue.^[^ ^16^, ^19^ ^]^ TFNA can carry microRNA or siRNA through simple sticky terminal binding.^[^ ^15^, ^20^ ^]^

Tannic acid (TA), a type of natural polyphenol, has attracted attention due to its anti‐inflammatory, proapoptotic, and antioxidant effects. Above all, polyphenols can interact with nucleic acids by hydrogen bonding, and this interaction is regulated by pH. TA can also mediate the size adjustment of FNANs.^[^ ^14^, ^21^ ^]^ To realize the intelligent loading/unloading of siRNA, increase the structural stability and control the size, we used the characteristics of TA to independently assemble TA, tFNA and siRNA into a controllable composite whole: STT. STT is similar in size to tFNA and enters the lysosome through endocytosis by caveolin.^[^ ^18^ ^]^ In the presence of lysosomal acid pH (5.5), the hydrogen bond is dissociated, thus releasing the TA‐coated siRNA‐tFNA (ST) complex. The simple sticky end was replaced with a DNA‒RNA hybridization sequence to respond to RNase H in the cytoplasm, thus disassembling ST into siRNA and pure tFNA. More importantly, tFNA can fully play a therapeutic role without destroying the structure.^[^ ^22^ ^]^ Previous studies by Lin et al. have shown that tFNA has good antioxidant, immune tolerance, and anti‐inflammatory properties.^[^ ^18^, ^23^ ^]^

**Figure 1:**
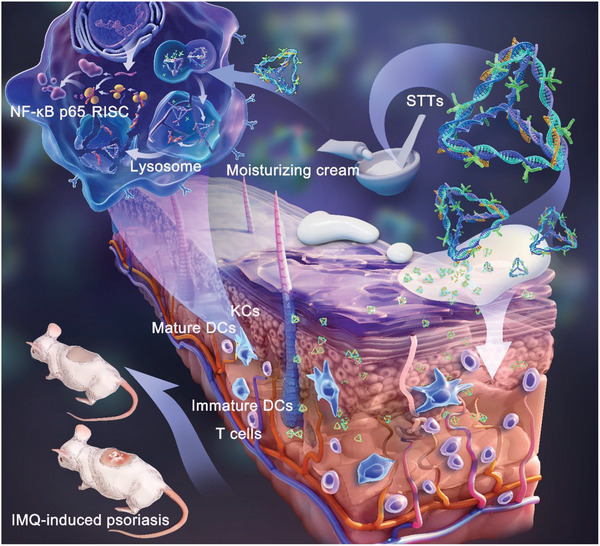
Schematic illustration showing the cascade‐responsive decomposition of STT and transcutaneous immunotherapy of psoriasis through silencing NF‐κB p65.

STT reduces the secretion of IL‐17, TNF‐α, and IL‐23 in the closed‐loop pathway by silencing NF‐κB p65. As shown in **Figure** **1**, STT can restore the normal immune environment of skin by inhibiting the excessive proliferation of KCs, reducing the large amounts of inflammatory factors secreted by DCs, and reducing the differentiation of mature DCs (mDCs). Therefore, the successful construction of STT provides a new idea and direction for siRNA delivery. The transdermal application of STT shows great potential in immune diseases, skin diseases and the delivery of immune vaccines.

## 2 Results and Discussion

### 2.1 Synthesis, Stability and Characterization of STT

**Figure 2:**
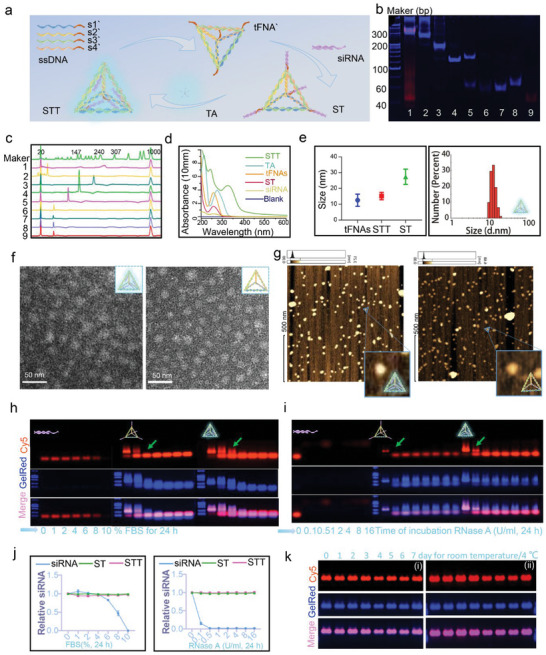
Synthesis, characterization, and stability of STT. (a) Schematic diagram showing the composition of ST and STT. (b) PAGE graph showing the successful synthesis of ST (1: ST, 2: tFNA`, 3: S1`+S2`+S3`, 4: S1`+S2`, 5: S4`, 6: S3`, 7: S2`, 8: S1` and 9: Cy5‐loaded siRNA). (c) CE graph showing the successful synthesis of ST (1: ST, 2: siRNA, 3: tFNA`, 4: S1`+S2`+S3`, 5: S1`+S2`, 6: S4`, 7: S3`, 8: S2`, and 9: S1`). (d) UV absorption spectroscopy of TA, siRNAs, tFNAs, STs and STTs. (e) Molecular size of tFNAs, STs and STTs measured by DLS. (f) TEM images of tFNAs and STTs. Scale bars are 50 nm. (g) AFM images of tFNAs and STTs. Scale bars are 25 nm and 200 nm. (h) Images of AGE showing Cy5 loaded siRNAs, STs and STTs after incubation with FBS in a concentration gradient (0%, 1%, 2%, 4%, 6%, 8%, and 10%) at 37°C for 24 h. (i) Images of AGE showing Cy5 loaded siRNAs, STs and STTs after incubation with RNase A at a concentration gradient (0, 0.1, 0.5, 1, 2, 4, 8, and 16 U/mL) at 37°C for 24 h. (j) Statistical chart showing the relative concentration of the undegraded siRNA shown in the images presented in (h) and (i). (k) Images of AGE showing Cy5 loaded STTs after incubation at room temperature and 4 °C for 0, 1, 2, 3, 4, 5, 6 and 7 days.

STT consists of internal ST and external TA (**Figure** **2**a). To enable the nanocarrier to assemble and unload siRNA freely, a DNA‒RNA hybridization sequence is used to respond to RNase H in the cytoplasm. Therefore, based on the synthesis rules of tFNAs,^[^ ^18b^ ^]^ single‐stranded DNA (ssDNA) with a DNA sticky end (ssDNA`) was used to combine siRNA with an RNA sticky end (Table S1, Supporting Information). Through a defined procedure (95 °C, 10 min; 4 °C, 20 min), four ssDNA`s were automatically assembled to form tFNA`. Next, siRNA and tFNA` (4:1) were mixed, left for 30 min at room temperature, and successfully assembled into ST. The results of polyacrylamide gel electrophoresis (PAGE) and capillary electrophoresis (CE) confirmed the successful synthesis of STs (Figure 2b and 2c and Figure S1a‐S1d, Supporting Information). For example, the increase in bp of ST relative to tFNA` is exactly the sum of the bp numbers of four siRNAs. The siRNA labeled with Cy5 displayed in red and DNA labeled with Gelred displayed in blue basically overlapped (Figure 2b), which more intuitively showed that siRNA was successfully loaded onto tFNA`. However, the smearing of siRNA was caused by the detachment of siRNA from STs owing to temperature rise in the PAGE experiment. Then, through simple mixing and vortexing, hydrogen bonds and π‐π accumulation between TA and nucleic acids can drive their binding.^[^ ^21^ ^]^ The successful assembly of STT was confirmed by UV scanning analysis. The peaks of TA (213 nm and 277 nm) and nucleic acid (258 nm) in the spectrum shifted significantly after the composition of STTs (243 nm and 324 nm), which is because the peak of catechol groups after the reaction was blueshifted (Figure 2d). Moreover, TA demonstrated good encapsulation efficiency in STT, indicating that TA binds extremely easily to nucleic acids (Figures S2, Supporting Information).

The key to using tFNA as a carrier is that its unique spatial structure and its 21 bp side length facilitate skin penetration.^[^ ^16^, ^19^ ^]^ Therefore, it is very important to keep STT consistent with tFNA in structure and size. Then, to verify the control of TA on the size of STT, dynamic light scattering (DLS) was used to determine the particle size of materials. We varied the concentration ratio of TA (0, 12.5, 25 to 50 µg/mL) and ST (1 µM). Figures S3a and S3b, Supporting Information, show that the particle size of STT gradually decreased with increasing TA concentration. The particle size of ST (27.40±4.86 nm) was higher than that of tFNA (12.6 ± 3.88 nm) because of the incorporation of siRNA. However, due to the presence of TA, the particle size of STT (15.20 ± 2.33 nm) was similar to that of tFNA (Figure 2e). TA adjust the size of nanomaterials, which is driven by hydrogen bonding and π‐π stacking.^[^ ^21^, ^24^ ^]^ ST, tFNA, and STT all presented negative charges (Figure S3c and S3d, Supporting Information). The higher negative charge of STT compared to that of ST also confirms the binding of TA and ST. Transmission electron microscopy (TEM) and atomic force microscopy (AFM) were further used to compare the size and shape of tFNA and STT. As shown in Figure 2f and 2g, both STT and tFNA form triangular structures, with uniform distributions and approximately similar sizes.

It is very important for nanomaterials to maintain overall structural stability and protect siRNA from enzyme and serum degradation before the release of siRNA. Agarose gel electrophoresis (AGE) was used to determine the stability of siRNA, ST and STT in fetal bovine serum (FBS) and RNase A (Figure 2h and 2i). Cy5 was used to label siRNA and determine the degradation degree of siRNA based on its fluorescence intensity. According to Figure 2j, within 24 h, siRNA alone was completely degraded in 10% FBS, while siRNA in ST and STT was hardly degraded. siRNA was completely degraded when the concentration of RNase A reached 0.5 U/mL, while siRNA in ST and STT did not degrade even in 16 U/mL RNase A within 24 h. Notably, by carefully observing the movement track of Cy5 (**green arrow**), we found that tFNAs in STs degraded more slowly than that in STTs, which can be attributed to the encapsulation effect of TA. In addition, the storage stability of STT was measured. STT can remain nondegradable at 4 °C and room temperature for one week, which is particularly important for the in vivo transdermal test (Figure 2k). Additionally. The degradation of siRNA, ST, and STT was observed over time when incubated in 10% FBS and 1 U/mL RNase A. As shown in Figures S4a and S4b, Supporting Information, the degradation of siRNA alone was the most rapid.

### 2.2 Transdermal Delivery, Controllable Release and Lysosomal Escape of the STTs

**Figure 3:**
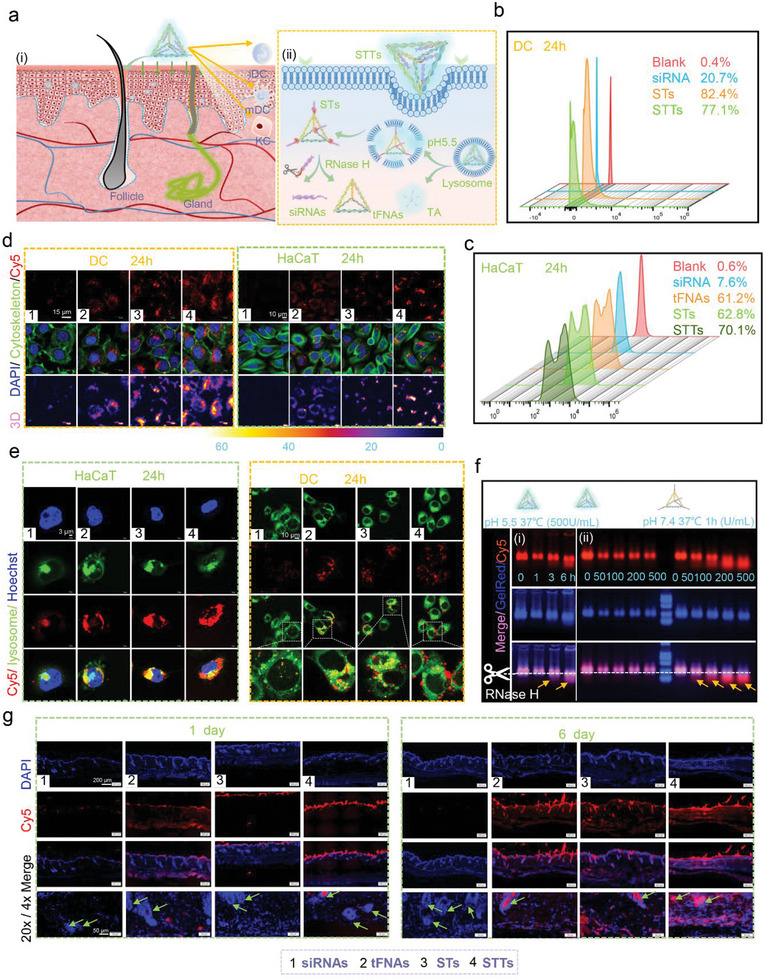
Transdermal delivery and responsive decomposition of STTs. (a) Schematic diagram showing the transdermal delivery and responsive decomposition of STTs. (b) and (c) Cellular uptakes of Cy5‐loaded siRNAs, STs and STTs in DCs and HaCaTs detected by flow cytometry. (d) Confocal fluorescence images showing the uptake of Cy5‐loaded siRNAs, tFNAs, STs, and STTs in DCs and HaCaTs for 24 h. (cytoskeleton: green; Cy5: red; nuclear: blue; 3D: 3D reconstruction of fluorescence microscopic images based on fluorescence intensity of Cy5). Scale bars are 15 µm and 10 µm. (e) Confocal fluorescence images showing the colocalization of Cy5‐loaded siRNAs, tFNAs, STs, STTs and lysosomes stained by Lysotracker in living DCs and HaCaTs after treatment for 24 h (Lysotracker: green; Cy5: red; Hoechst: blue). Scale bars are 3 µm and 10 µm. (f) i: Images of AGE showing the decomposition of Cy5‐loaded STTs under 500 U/mL RNase H for 0, 1, 3, and 6 h at pH 5.5. ii: Images of AGE showing the decomposition of Cy5‐loaded STs and STTs under RNase H in a concentration gradient (0, 50, 100, 200 and 500 U/mL, pH: 7.4) for 1 h at 37 °C. (g) Confocal fluorescence images showing the transdermal delivery of Cy5‐loaded siRNAs, tFNAs, STs, and STTs for 1 and 6 days. Scale bars are 200 µm and 50 µm.1: siRNAs, 2: tFNAs, 3: STs, 4: STTs.

STT is designed to penetrate through the skin into a variety of immune cells, especially KCs and DCs (**Figure** **3**a). KCs are involved in controlling the physiological barrier of the skin, and both AD and psoriasis are related to skin barrier damage.^[^ ^6^ ^]^ Moreover, KCs are targets for effector cytokines (TNF‐α and IL‐17). KCs increase the synthesis of effector cytokines, thereby inducing T cells, DCs, and other immune cells to enter the injured site to activate innate immunity^[^ ^6^, ^25^ ^]^ As DCs are antigen‐presenting cells, DC activation controls the activation and development of immune responses. TFNA as a carrier has successfully delivered microRNA, small molecule compounds, siRNA, etc., into cells.^[^ ^15^, ^18^, ^20^ ^]^ Therefore, flow cytometry and immunofluorescence techniques were used to observe whether STTs can be successfully taken up by HaCaTs and DCs. Cy5 in the tFNA group was modified on ssDNA, while Cy5 in the other groups was modified on siRNA. The entry efficiency of siRNA (HaCaTs: 20.7%, DCs: 7.6%) was much lower than that of STTs (HaCaTs: 77.1%, DCs: 70.1%) (Figure 3b and 3c). There was no significant difference in the efficiency of STs and STTs. To further confirm that tFNAs can help siRNAs enter the cell, we conducted a visual observation of the entry situation. Whether in DCs or HaCaTs, the fluorescence intensity of Cy5 of STTs was much higher than that of siRNAs (Figure 3d).

Another difficulty with transdermal gene delivery is that gene delivery, which involves entering cells through endocytosis, usually delivers the cargo into a low pH endosome or lysosome for degradation.^[^ ^12^ ^]^ Therefore, STT is are designed to wrap around ST with TA, and the ester bonds in the TA structure can degrade in acidic environments.^[^ ^14^, ^21^ ^]^ The degradation of TA causes the decomposition of STT and the release of TA and ST (Figure 3a). Colocalization analysis was performed to verify the lysosomal escape of STTs in DCs and HaCaTs. Cy5‐loaded STTs, STs, tFNAs, and siRNAs were incubated with DCs and HaCaTs for 24 h, and then the fluorescence of Lysotracker and Cy5 was visually observed. The green LysoTracker and the red Cy5 display yellow fluorescence after colocalization. Only a small portion of siRNAs alone entered the cell, and Cy5 fluorescence basically overlapped with Lysotracker fluorescence. The Cy5 fluorescence distribution areas of tFNAs and STs were higher than that of siRNAs, but only a small portion of Cy5 did not overlap with LysoTracker. In contrast, only a small portion of Cy5 in STTs overlapped with the LysoTracker signal (Figure 3e).

The pH‐responsive decomposition is the basis of lysosomal escape. Furthermore, the acid‐responsive reaction of STTs was explored by AGE. As shown in Figure 3f (i), the gel diagram of STTs after being maintained for 0, 1, 3, and 6 h in an RNase H solution (500 U/mL, pH 5.5) shows the decomposition of STTs. Starting at 3 h, STTs showed significant decomposition. However, under normal pH conditions (pH: 7.4), STTs did not decompose (Figure S5, Supporting Information).To more accurately confirm that the decomposition of STTs originated from TA, further AGE analysis was performed. STTs and STs were incubated with RNase H for 1 h in pH 7.4. When the concentration of RNase H reached 50 U/mL, STs began to decompose (yellow arrow), while STTs did not decompose even in 500 U/mL RNase H (Figure 3f (ii)). Therefore, the decomposition of STTs originated from the decomposition of TA in acidic environments. At normal pH, the RNase H‐responsive sequences of STTs will not be directly exposed to RNase H under the wrapping of TA, so STTs will not undergo decomposition.

Based on these previous results, STT exhibits excellent serum stability, enzyme stability, and storage stability for one week (Figure 2h‐2k). STT has also been proven to have excellent cellular uptake capabilities, and our designed nanodelivery vectors have successfully solved the low cellular uptake rate of siRNA. In addition, TA can not only participate in regulating the size of STT to make it more easily absorbed by cells and tissues, similar to vector tFNA, but form a layer of protection against siRNA detachment from STT before acid‐responsive decomposition. Moreover, ST dissociated from STT was successfully decomposed into siRNA and tFNA in the cytoplasm under the action of RNase H. Finally, the successful transdermal delivery of STT was verified through frozen sections of skin tissue. Consistent with the results of the cell uptake experiment, STT was widely distributed in the surface layer of the skin in one day, while by 6 days, STT was present in all layers of the skin. (Figure 3g). Interestingly, the amount of fluorescence deposition of STT was greater than that of ST, possibly because the size of STT is more similar to that of tFNA.

In summary, STT has the advantages of intelligent and controllable loading/unloading of nano‐transdermal drugs. STT can be considered a stable, efficient and intelligent siRNA transdermal delivery system that can quickly penetrate DCs, HaCaTs, and skin tissues, avoiding the disadvantages of traditional siRNA delivery.

### 2.3 Specific NF‐κB Silencing by STTs in HaCaTs Inhibits Inflammation

NF‐κB signals play a role in a wide range of inflammatory processes and are associated with psoriasis susceptibility.^[^ ^3^, ^7^, ^26^ ^]^ Both toll‐like receptor (TLR) and TNF receptor (TNFR) can participate in activating NF‐κB, thereby affecting psoriasis.^[^ ^3^, ^27^ ^]^ The inhibition of NF‐κB can be observed in most cured psoriasis patients.^[^ ^3^ ^]^ The onset of psoriasis begins with the activation of innate immunity and is subsequently amplified by acquired immunity. KCs provide a source of mediators for innate immunity.^[^ ^28^ ^]^ One hypothesis is that trauma stimulates damage to KCs, thereby stimulating DCs to activate and secrete related inflammatory factors, thereby enhancing the development of Th17 cells.^[^ ^27^, ^29^ ^]^ The activation and transmission of NF‐κB signals in KCs are essential for the development of psoriasis. HaCaT, a KC line, was selected to verify the silencing effect of STTs on NF‐κB signaling in vitro.

**Figure 4:**
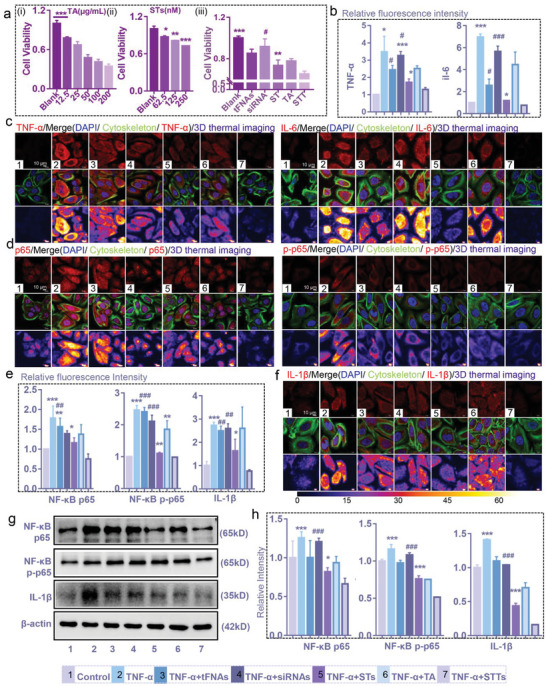
STTs reduced TNF‐α‐induced inflammation in HaCaTs through inhibiting NF‐κB p65. (a) Statistical chart of CCK‐8 showing the cell viability of TNF‐α‐induced HaCaTs under the treatment of TA (i: 0, 12.5, 25, 50, 100, and 200 µg/mL), STs (ii: 0, 62.5, 125, and 250 nM), and other drugs (iii: 250 nM tFNAs, STs, STTs, 1000 nM siRNAs and 12.5 µg/mL TA) (n = 3). Statistical analysis: *P < 0.05, **P < 0.01, ***P < 0.001, #P < 0.05, ##P < 0.01, ###P < 0.001. ii *: Blank group versus other groups; iii *: STT group versus other groups, #: ST group versus other groups. (b) The quantitative analysis of TNF‐α and IL‐6 in HaCaTs after different treatment for 24 h based on the immunofluorescence images (n = 3). (c) Immunofluorescence images of TNF‐α and IL‐6 in HaCaTs after different treatment for 24 h (cytoskeleton: green; nucleus: blue; TNF‐α and IL‐6: red; 3D thermal imaging: 3D reconstruction of fluorescence microscopic images based on fluorescence intensity of TNF‐α and IL‐6). Scale bars are 10 µm. (d) Immunofluorescence images of NF‐κB p65 and NF‐κB p‐p65 in HaCaTs after different treatment for 24 h. (cytoskeleton: green; nucleus: blue; NF‐κB p65 and NF‐κB p‐p65: red; 3D thermal imaging: 3D reconstruction of fluorescence microscopic images based on fluorescence intensity of NF‐κB p65 and NF‐κB p‐p65). Scale bars are 10 µm. (e) The quantitative analysis of NF‐κB p65, NF‐κB p‐p65 and IL‐1β in HaCaTs after different treatment for 24 h based on fluorescence microscopic images(n = 3). (f) Immunofluorescence images of IL‐1β in HaCaTs after different treatment for 24 h. (cytoskeleton: green; nucleus: blue; IL‐1β: red; 3D thermal imaging: 3D reconstruction of fluorescence microscopic images based on fluorescence intensity of IL‐1β). Scale bars are 10 µm. (g) WB analysis of the NF‐κB p65, NF‐κB p‐p65 and IL‐1β expression level. (h) The relative protein expression intensity NF‐κB p65, NF‐κB p‐p65 and IL‐1β in HaCaTs after different treatment for 24 h (n = 3). 1: Control, 2: TNF‐α, 3: TNF‐α+tFNAs, 4: TNF‐α+siRNAs, 5: TNF‐α+STs, 6: TNF‐α+TA, 7: TNF‐α+STTs; β‐actin was used as an internal control. Statistic differences are significant between the two groups (p < 0.05). Statistical analysis: *P < 0.05, **P < 0.01, ***P < 0.001, #P < 0.05, ##P < 0.01, ###P < 0.001. *: STT group versus other groups, #: ST group versus other groups.

First, we tested the biological safety of STTs. Through experimental verification with CCK‐8, appropriate concentrations of TA (12.5 and 25 µg/mL) were selected that had no cytotoxicity to normal HaCaTs but did exhibit has cytotoxicity to TNF‐α‐induced HaCaTs (Figure S6a, Supporting Information). In addition, at concentrations of 62.5, 125, and 250 nM, STs had no effect on the cell viability of HaCaTs (Figure S6b, Supporting Information), while STs and TA both inhibited the cell viability of TNF‐α‐induced HaCaTs in a concentration‐dependent manner (**Figure** **4**a). Based on the previous analysis of particle size and encapsulation efficiency, the encapsulation efficiency of 50 µg/mL TA and 1 µM STs binding peaked, and the particle size of the composite was closest to that of tFNA. Finally, 250 nM STTs containing 12.5 µg/mL TA and 250 nM STs was used in the next experiments. Considering that the foundation of psoriasis is the excessive proliferation of KCs, the inhibitory effect of STTs on the proliferation of inflammatory HaCaTs is important. Figure 4a shows that STTs had a better inhibitory effect on the activity of TNF‐α‐induced HaCaTs than STs.

The activation of NF‐κB, as a key event downstream of TNF signaling, not only induces inflammation but also affects the behavior of the affected cells, such as activation, maturation, proliferation, migration, and survival.^[^ ^3^, ^30^ ^]^ We previously demonstrated the effect of STTs on proliferation and further determined the impact of STTs on migration. STTs inhibited inflammatory HaCaT migration, consistent with the migration of HaCaTs without inflammatory stimulation (Figure S6c, Supporting Information). In addition, the measurement of cell apoptosis revealed that STTs had a certain proapoptotic effect on TNF‐α‐induced HaCaTs (Figure S6d, Supporting Information). NF‐κB inhibitors and siRNAs are also commonly used to inhibit tumor apoptosis and migration. In particular, knockdown of NF‐κB p65 causes downregulation of apoptosis suppression genes.^[^ ^31^ ^]^ In addition, the activation of NF‐κB p65 often promotes cell proliferation and reduces apoptosis, while inhibition NF‐κB p65 to promote apoptosis of inflammatory KCs.^[^ ^32^ ^]^ The simultaneous presence of TNF‐α and TNF‐like weak inducer of apoptosis (TWEAK) can induce apoptosis, but the expression of TWEAK in unstimulated and stimulated KCs is low and insufficient to cause apoptosis. Inhibiting the expression of NF‐κB makes HaCaTs more sensitive to TNF‐α and causes HaCaTs to undergo apoptosis.^[^ ^33^ ^]^ Therefore, the cytotoxicity and proapoptotic effects of STs and STTs on inflammatory HaCaTs are attributed to the knockdown of NF‐κB p65 by siRNA. These results preliminarily verified the inhibitory effects of STTs on the migration, proliferation and survival of inflammatory HaCaTs, but the increased expression of inflammatory factors in HaCaTs is more critical for subsequent immune activation and the progression of psoriasis.

KCs are the direct and key target of TNF signal transduction in human psoriasis.^[^ ^6^, ^27^ ^]^ Anti‐TNF therapy is also one of the most effective methods for treating psoriasis.^[^ ^3^, ^28^ ^]^ Inhibition of NF‐κB can successfully interfere with the signal transduction of TNF‐TNFR, thereby altering skin inflammation. Many psoriasis‐related genes are also associated with the TNF‐NF‐κB signal transduction pathway.^[^ ^3^, ^34^ ^]^ The monitoring of TNF signals is crucial for the maintenance of normal physiological functions, as well as for tissue regeneration and host defense. Therefore, exploring the effect of silencing NF‐κB p65 on HaCaT inflammation is important for better understanding the pathogenesis of psoriasis. Dysfunctional keratinocytes overexpressing IL‐6, TNF‐α, and IL‐1β can promote the differentiation of DCs and amplify inflammation.^[^ ^32b^ ^]^ As shown in Figure 4b, 4c, Figure S7a and S7b, Supporting Information, after STT processing, the expression of TNF‐α and IL‐6 in HaCaTs was reduced at both the gene and protein levels, and the effects of STs and STTs significantly differed. Furthermore, we verified the silencing effect of STT on NF‐κB p65, and the transport of p65 from the cytoplasm to the nucleus after activation by TNF‐α was visually observed by immunofluorescence. Single siRNAs exhibit a low silencing effect due to its low entry efficiency. Compared to STs, STTs exhibited significantly stronger gene and protein inhibition of NF‐κB p65 and phosphorylated NF‐κB p65 (NF‐κB p‐p65) (Figure 4d, 4e, 4g, 4h and Figure S7c, Supporting Information). In addition, STTs reduced the expression of IL‐1β (Figure 4f‐4g and Figure S7d, Supporting Information), which is also necessary for maintaining the normal function of KCs.^[^ ^35^ ^]^

Based on our research, STT can exhibit a more prominent silencing effect on NF‐κB p65 than ST through lysosomal escape and the synergistic anti‐inflammatory effect of TA, thereby inhibiting the expression of IL‐1β, IL‐6, and TNF‐α. STT is a prerequisite for blocking the subsequent inflammatory signals transmitted by HaCaTs to DCs by controlling the inflammatory expression of HaCaTs, which helps to avoid DC differentiation and inflammation amplification.

### 2.4 Specific Silencing of NF‐κB by STT Inhibits Inflammation and Maturation of DCs

**Figure 5:**
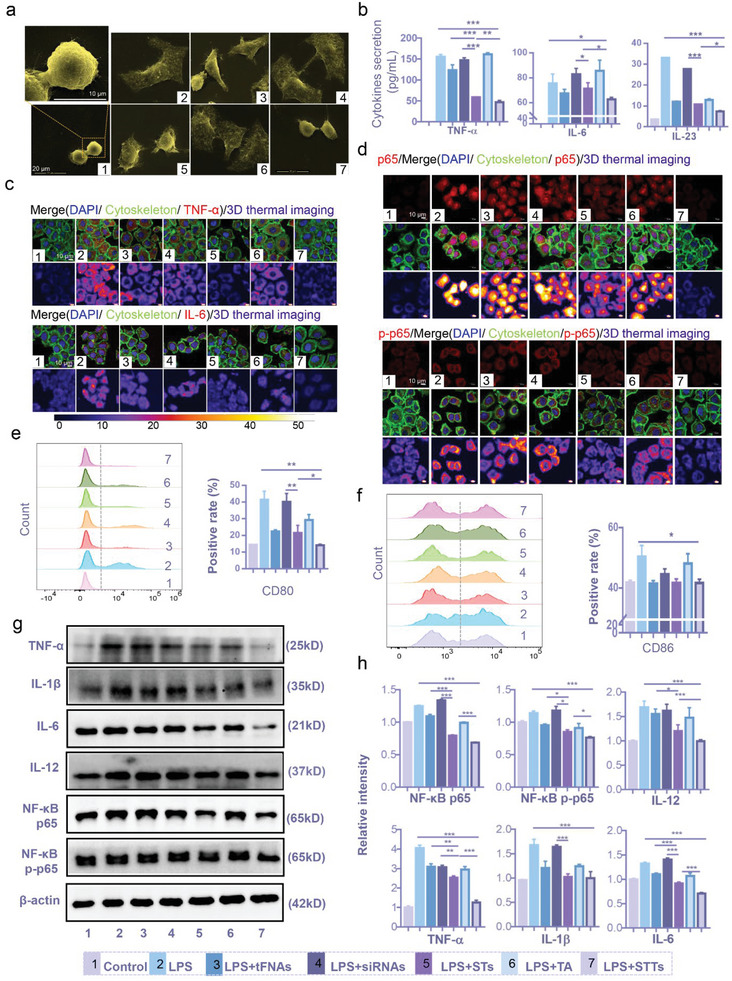
STTs reduced inflammation and maturation of DCs through inhibiting the expression of NF‐κB. (a) SEM images showing the morphological changes of DCs under different treatments. Scale bars are 10 µm and 20 µm. (b) ELISA detection of TNF‐α, IL‐6 and IL‐23 in DCs after different treatment for 24 h (n = 3). (c) and (d) Immunofluorescence images of TNF‐α, IL‐6, NF‐κB p65 and NF‐κB p‐p65 in DCs after different treatment for 24 h. (cytoskeleton: green; nucleus: blue; TNF‐α, IL‐6, NF‐κB p65 and NF‐κB p‐p65: red; 3D thermal imaging: 3D reconstruction of fluorescence microscopic images based on fluorescence intensity of TNF‐α, IL‐6, NF‐κB p65 and NF‐κB p‐p65). Scale bars are 10 µm. (e) Flow cytometry images and statistical analysis showed the changes of DCs labeled with CD80 (n = 3). (f) Flow cytometry images and statistical analysis showed the changes of DCs labeled with CD86 (n = 3). (g) WB analysis of the NF‐κB p65, NF‐κB p‐p65, TNF‐α, IL‐6, IL‐12 and IL‐1β expression level. (h) The relative protein expression intensity of NF‐κB p65, NF‐κB p‐p65, TNF‐α, IL‐6, IL‐12 and IL‐1β in DCs after different treatment for 24 h (n = 3). 1: Control, 2: LPS, 3: LPS+tFNAs, 4: LPS+siRNAs, 5: LPS+STs, 6: LPS+TA, 7: LPS+STTs; β‐actin was used as an internal control. Statistic differences are significant between the two groups (p < 0.05). Statistical analysis: *P < 0.05, **P < 0.01, ***P < 0.001.

In the inflammatory microenvironment of psoriatic skin, the activation of immunostimulatory DCs is highly common.^[^ ^36^ ^]^ Lou et al. demonstrated that the culture supernatant of KCs can activate inflammatory DCs.^[^ ^4^, ^37^ ^]^ In addition to the aforementioned TNF‐α and IL‐23/IL‐17A is also a topic of high interest in the study of cytokines involved in the pathogenesis of psoriasis.^[^ ^35^, ^38^ ^]^ The activation of the NF‐κB is closely related to the elevation of IL‐17A.^[^ ^39^ ^]^ In addition, TNF‐α indirectly enhances the effect of IL‐17 on histiocytes, thereby directly exacerbating psoriasis lesions.^[^ ^29^, ^40^ ^]^ DCs have the highest expression of IL‐23 in the skin, and the IL‐23/IL‐17 axis is believed to be caused by the activation of DCs in the skin to produce IL‐23, which ultimately acts on KCs through IL‐17.^[^ ^6^ ^]^ As the strongest antigen‐presenting cell, DCs play an important role in the progression of psoriasis, especially in the innate immune system. Mature myeloid DCs activate T cells to participate in innate immunity by secreting the costimulatory molecules CD80 and CD86 and promote the excessive proliferation of KCs by secreting IL‐12, IL‐23, and TNF‐α for activation.^[^ ^25^, ^41^ ^]^ Inhibiting DC activation and maturation can successfully inhibit the progression of psoriasis.^[^ ^25^ ^]^ Hence, bone marrow dendritic cells (BMDCs) or DC line stimulated with 100 ng/mL LPS were chosen to establish the inflammatory model of DCs. As shown in **Figure** **5**a, mDCs induced by LPS exhibit enlarged cytoplasm and irregular protrusions in cell morphology, while DCs treated with STTs approach immature DCs (iDCs), exhibiting smaller circular shapes with almost no protrusions. The cell supernatant of BMDCs after 24 h of drug action was collected to determine important inflammatory factors. IL‐6 and the most important cytokine TNF‐α and IL‐23 secretion were measured. Inflammatory cytokines were significantly increased after LPS stimulation, while siRNAs alone had little effect on their secretion. After treatment with tFNAs and TA, there was almost no significant reduction in cytokines. STs significantly reduced the secretion of three cytokines, but to a lesser extent than STTs (Figure 5b). STTs reshape the microenvironment of psoriasis by reducing the secretion of IL‐6 and TNF‐α and reduces Th17 cell activation by inhibiting IL‐23 secretion.^[^ ^36^ ^]^

In addition, the expression of TNF‐α and IL‐6 in DCs reconfirmed the inhibitory effect of STTs on inflammatory factors (Figure 5c and Figure S8, Supporting Information). NF‐κB is a key regulatory signal for IL‐17 and IL‐23 and participates in the mediation of the TNF‐TNFR signaling pathway.^[^ ^3^, ^7^, ^34^ ^]^ Furthermore, the silencing effect of STTs on NF‐κB p65 was determined through quantitative analysis of proteins. After LPS treatment, the activation of NF‐κB p65 and nuclear transport phenomena can be clearly observed. The expression of NF‐κB p65 in the STT group was consistent with that in the control group. The fluorescence intensity was significantly lower than that of the LPS‐stimulated group. Similarly, the expression of NF‐κB p‐p65 returned to normal levels after STT treatment (Figure 5d and Figure S8, Supporting Information). We reasonably believe that STTs reduce IL‐23 and TNF‐α expression via inhibition of the NF‐κB signaling pathway.

The previously studied inflammatory factors are secreted after differentiation from iDCs to mDCs, so the mature state of DCs was further verified. DCs can be labeled with MHC II‐PE and CD11c‐FITC, while CD86‐PE‐Cy7 and CD80‐APC are used to label mDCs (Figure S9, Supporting Information). As expected, the number of CD80‐labeled cells after ST treatment (21.6 ± 2.542%) was significantly higher than that after STT treatment (14.17 ± 0.3383%), but both were significantly different from the number of LPS‐induced DCs (41.53 ± 2.864%) (Figure 5e). The numbers of DCs labeled with CD86‐PE‐Cy7 showed that the effects of STs and STTs were not different and were consistent with respect to the control group, and both significantly inhibited LPS‐induced DC activation (Figure 5f).

Finally, the previous results were confirmed again through western blotting (WB). Treatment with STTs caused IL‐1β, IL‐12, IL‐6, and TNF‐α secretion by DCs in innate immunity to decrease, which was achieved by silencing and decreasing the expression of NF‐κB p65 (Figure 5g and 5h).

### 2.5 Treatment of IMQ‐Induced Psoriasis with STTs

**Figure 6:**
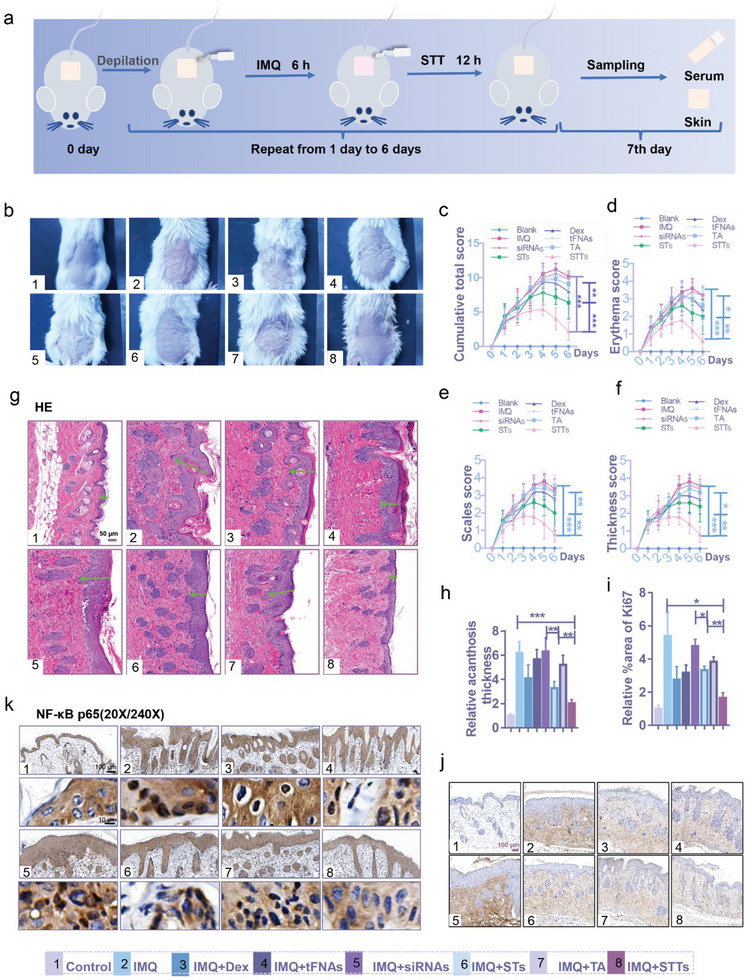
Therapeutic effect of STTs on IMQ‐induced psoriasis in mice. (a) Schematic diagram of IMQ‐induced psoriasis in mice. (b) General views of IMQ‐induced psoriasis in mice after treatment with Dex, siRNAs, tFNAs, STs, TA, and STTs for 6 days. (c)‐(f) The cumulative total score, erythema score, scales score, and thickness score change of IMQ‐induced psoriasis in mice after treatment with Dex, siRNAs, tFNAs, STs, TA, and STTs for 0, 1, 2, 3, 4, 5, and 6 days based on PASI rules. (n = 5). (g) Representative histopathological images of skin sections from normal and IMQ‐induced mice treated with Dex, siRNAs, tFNAs, STs, TA, and STTs on days 6 according to HE images (thickness of skin epidermis: green arrow). Scale bars are 50 µm. (h) Statistical analysis showing the thickness of the skin epidermis according to HE images (n = 5). (i) Representative IHC images of skin sections from normal and IMQ‐induced mice treated with Dex, siRNAs, tFNAs, STs, TA, and STTs on days 6 according to Ki67 staining. Scale bars are 100 µm. (j) Statistical analysis showing the positive area changes of Ki67 according to IHC images (n = 5). (k) Representative IHC of skin sections from normal and IMQ‐induced mice treated with Dex, siRNAs, tFNAs, STs, TA, and STTs on days 6 according to NF‐κB p65 staining. Scale bars are 100 µm and 10 µm. 1: Control, 2: IMQ, 3: IMQ+Dex, 4: IMQ+tFNAs, 5: IMQ+siRNAs, 6: IMQ+STs, 7: IMQ+TA, 8: IMQ+STTs; β‐actin was used as an internal control. Statistic differences are significant between the two groups (p < 0.05). Statistical analysis: *P < 0.05, **P < 0.01, ***P < 0.001.

Psoriasis, a recurrent and incurable skin disease, requires long‐term treatment, and local treatment is preferred.^[^ ^5^ ^]^ Although the barrier structure of the skin creates obstacles for drug transportation, previous results have successfully confirmed that STT has good transdermal efficiency (Figure 3). Here, an in vivo fluorescence imaging system (IVIS) was used to determine the transdermal efficiency, as shown in Figure S10, Supporting Information. The result was similar to previous findings, that is, the Cy5 signals of STTs and STs were much higher than that of siRNAs. The reduction in TNF‐α and IL‐23/IL‐17 inflammatory factor secretion, as key therapeutic targets, will inhibit the development of psoriasis.^[^ ^28^, ^29^, ^39^ ^]^ Imiquimod (IMQ)‐induced psoriasis in mice was used to explore whether STTs can change the inflammatory microenvironment of psoriasis by suppressing the expression of NF‐κB p65. Skin inflammation caused by IMQ generally peaks on days 4–5, and a highly inflammatory state continues within 6–7 days. As shown in **Figure** **6**a, 80 mg of medical IMQ cream (1 mg/cm^2^, 2 cm * 2 cm) was applied to the skin every day. After 6 h, the skin was washed and coated with Vaseline cream containing 100 µL STTs (STs: 1 µM, TA: 50 µg/mL) and covered with a medical patch. In addition, in the local treatment of psoriasis, glucocorticoids are widely considered the first‐line anti‐inflammatory treatment.^[^ ^42^ ^]^ Therefore, cream containing 90 µg of dexamethasone (Dex) was selected as a positive drug control group.^[^ ^43^ ^]^ After IMQ treatment, the skin began to show significant erythema, wrinkles, desquamation, and skin thickening the next day. The severity of skin inflammation was recorded with the psoriasis area and severity index (PASI) for 6 consecutive days. After IMQ application, the PASI score increased rapidly and peaked on the fifth day. STTs significantly reduced the formation of erythema and desquamation beginning on the fourth day, and there was a significant difference between the skin score of the STT group and that of the ST group on the sixth day (Figure 6b‐6f). As shown in Figure 6b, the skin of the STT‐treated group was very similar to the skin of the control group. The other groups had significant skin wrinkles and thickening. Then, the skin of the mice was stained with hematoxylin and eosin (HE). From the skin structure, it can be seen that STT treatment reduced acanthosis, rod‐shaped ridges, hyperkeratosis, and epidermal thickening (Figure 6g). Further quantitative analysis of the thickness of the epidermis showed that the skin thickness after STT treatment was much thinner than that of the IMQ group (Figure 6h).

The main pathological manifestation of psoriasis is excessive proliferation of KCs. The activation of epidermal KCs is indispensable in the initiation, development and regulation of skin inflammation, such as psoriasis and AD.^[^ ^6^ ^]^ Ki67, a cell proliferation‐related antigen, reflects the degree of proliferation of KCs and the severity of psoriasis.^[^ ^44^ ^]^ Immunohistochemical (IHC) staining was applied to the epidermis with anti‐Ki67 to analyze KC proliferation. When NF‐κB p65 was blocked, a significant decrease in the proliferative activity of KCs was observed in the ST and STT groups, and the effect of STT was more profound than that of ST. (Figure 6i and 6j).

**Figure 7:**
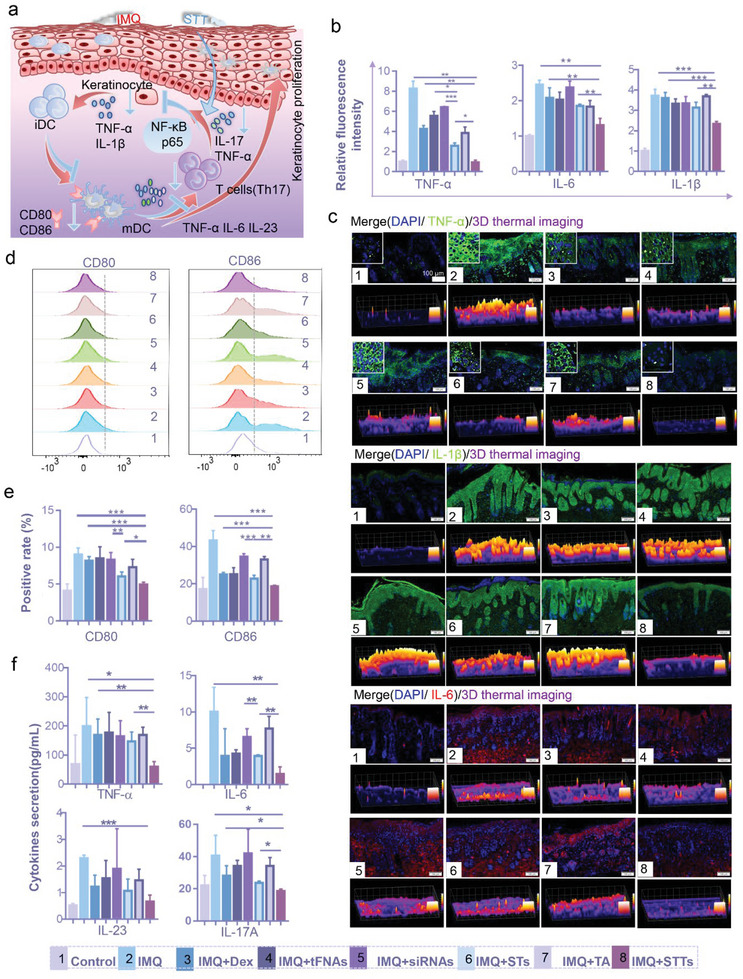
Anti‐inflammation and anti‐maturation of DCs by STTs on IMQ‐induced psoriasis. (a) Schematic diagram showing the mechanisms of inflammation induced by IMQ in mice (orange arrow) and the mechanisms of anti‐inflammation progression of STTs (blue arrow) in psoriasis. (b) and (c) Immunofluorescence images and its quantitative analysis of TNF‐α, IL‐1β and IL‐6 in skin tissues after different treatment for 6 days (cytoskeleton: green; nucleus: blue; TNF‐α, IL‐1β and IL‐6: red; 3D thermal imaging: 3D reconstruction of fluorescence microscopic images based on fluorescence intensity of TNF‐α, IL‐1β and IL‐6; n = 5). Scale bars are 100 µm. (d) and (e) Flow cytometry images and its statistical analysis showing the changes of DCs labeled with CD80 and CD86 (n = 4). (f) ELISA detection of TNF‐α, IL‐6, IL‐17A and IL‐23 in serum of psoriatic mice after different treatment for 6 days (n = 3). 1: Control, 2: IMQ, 3: IMQ+Dex, 4: IMQ+tFNAs, 5: IMQ+siRNAs, 6: IMQ+STs, 7: IMQ+TA, 8: IMQ+STTs; β‐actin was used as an internal control. Statistic differences are significant between the two groups (p < 0.05). Statistical analysis: *P < 0.05, **P < 0.01, ***P < 0.001.

The mechanism of STTs in treating psoriasis is to silence the NF‐κB p65 gene, which affects its transcription and ultimately leads to NF‐κB p65 protein degradation. In vitro studies have confirmed that STTs can successfully suppress NF‐κB p65 signaling in DCs and HaCaTs. Therefore, analyzing NF‐κB p65 in psoriatic mice is essential to explain the therapeutic mechanisms of STTs. The IHC images of NF‐κB p65 reveal that after IMQ successfully induces psoriasis, NF‐κB p65 is transported to the nucleus, presenting high nuclear expression. Only after using STTs was a significant decrease in p65 expression observed (Figure 6k). Two of the most important cytokine axes in psoriasis, TNF‐α and IL23/IL17, are connected by the NF‐κB signaling pathway. TNF‐α participates in the occurrence of numerous events through TNFR. The activation of NF‐κB promotes inflammation.^[^ ^25^ ^]^ In animals, TNFR induces activation of the NF‐κB signaling pathway to activate DCs resident in the skin to produce IL‐23, resulting in activation of the IL‐23/IL‐17 axis, which is also necessary for the development of psoriasis.^[^ ^6^ ^]^ Therefore, as shown in **Figure** **7**a, we hypothesize that the stimulation of KCs by IMQ causes KCs to produce large amounts of TNF‐α and IL‐1β, and TNF‐α participates in activating the NF‐κB signaling pathway, which causes DCs to secrete more inflammatory factors, especially TNF‐α and IL‐23. IL‐23 activates downstream Th17 cells to secrete IL‐17, which in turn acts on KCs to cause abnormal proliferation (orange arrow). STTs interrupt the transmission of the inflammatory axis initially caused by TNF‐α by silencing and inhibiting the expression of NF‐κB p65 protein, ultimately reducing the levels of pathogenic cytokines (blue arrow).

First, the expression of several common inflammatory factors was measured. As shown in Figure 7b and 7c, treatment with STTs resulted in significant decreases in the expression of IL‐6, IL‐1β, and TNF‐α in the skin tissue of psoriatic mice. The decline in TNF‐α was especially noticeable. Dex, a commonly used drug in clinical practice, had no prominent effect compared to STTs. The mDCs rather than iDCs activate T cells and participate in the activation of the IL‐23/IL‐17 axis by secreting the costimulatory molecules CD80 and CD86.^[^ ^25^ ^]^ The maturity of DCs was further analyzed. The expression of CD80 and CD86 in DCs in spleen cells was determined by flow cytometry. CD45 was used to label white blood cells, and MHC II and CD11c were used to label DCs to analyze the number of cells expressing CD80 and CD86‐positive DCs (Figure S9, Supporting Information). As shown in Figure 7d and 7e, the maturation of DCs after treatment with STTs was almost identical to the normal level, and there was a statistically significant difference in the therapeutic effects of STTs and STs. The positive cell rates of CD80 and CD86 reflected the inhibition of DC activation after STT treatment. Finally, quantitative analysis of inflammatory factors in the serum of mice was conducted to verify again that the NF‐κB pathway was blocked. The levels of IL‐6, IL‐23, IL‐17, and TNF‐α in serum were consistent with the predictions. The levels of inflammatory factors in psoriasis mice and mice treated with siRNAs alone were almost the highest. The effects of other drugs are not very stable, and overall, they slightly reduced the expression of inflammatory factors. Both STs and STTs stably reduced the secretion of inflammatory factors, and STTs had a better effect (Figure 7f). In summary, we have confirmed that STT, a transdermal gene drug that can accurately release siRNA, exhibits a significant silencing effect on NF‐κB p65 in mouse psoriasis dermatitis after IMQ stimulation. The blocking of NF‐κB p65 by STT inhibits the activation of DCs, thereby weakening the inflammatory transmission between TNF‐α and the IL‐23/IL‐17 axis, which ultimately restores the microenvironment of psoriasis to normal.

## 3 Conclusion

Transdermal gene therapy is very promising for diseases caused by the destruction of skin barriers and immune disorders. We synthesized pH‐responsive composite‐structure STT that can intelligently load/unload siRNA for the treatment of psoriasis. The overall structure is wrapped by natural polyphenol TA to prevent lysosomal degradation and trigger an acid response to accurately release ST. The specific DNA and RNA hybridization sequences that respond to RNase H in the cytoplasm cause siRNA and tFNA to separate into independent molecules and function independently. As a transdermal RNAi drug, STT not only has good transdermal and cellular uptake efficiency but also ensures stability during siRNA delivery. STT specifically silences and inhibits the transmission of NF‐κB signals and inhibits the maturation of DCs, thereby effecting changes in the expression of the key cytokines TNF‐α and IL‐23/IL‐17, which ultimately reduces the proliferation of KCs. The treatment of psoriasis by STT basically involves the innate and acquired immunity of the skin, which plays a powerful role in maintaining the immune homeostasis of the skin. The emergence of STT provides new prospects for vaccination, wound healing, and the treatment of skin cancer and other skin diseases.

## 4 Experimental Section

### Synthesis of ST and STT

tFNA and tFNA` were synthesized separately according to the previously reported rules for tFNA preparation. Four ssDNA (S1‐S4) and ssDNA` (S1`‐S4`) were formed into a system with TM buffer (10 mM Tris‐HCl and 1 mM MgCl_2_, pH = 8.0). Then, during denaturation (95°C, 10 min) and recombination (4°C, 20 min), the DNA in the system self‐assembled into tFNA and tFNA`. Then, 4000 nM siRNA was mixed with 1000 nM tFNA` and incubated at room temperature for 30 min. Based on the principle of base complementary pairing, the sticky ends of RNA in siRNA bound to the four sticky ends of DNA in tFNA` to form STs. Next, TA (12.5 µg/mL–200 µg/mL) dissolved in enzyme‐free water in advance was added to 1000 nM ST solution separately and vortexed for several seconds to ensure sufficient mixing. The mixed solution was finally centrifuged to obtain precipitate STT. The amount of unbound TA was quantified by measuring the absorbance of TA at 278 nm using a Nanophotometer N60 (IMPLEN, Germany). The encapsulation efficiency of TA in STs was examined as follows.

### Characterization of STT

CE and 8% PAGE were used to verify the synthesis of tFNA and ST. Among the PAGE results, siRNA was labeled with Cy5, GelRed staining was used to label nucleic acids, and the overlap of the Cy5 and GelRed channels confirmed the successful binding of siRNA and tFNA`. In addition, the IMPLEN Nanophotometer N60 was used to determine the UV‒Vis absorption spectra of TA, tFNA, siRNA, ST and STT. The successful binding of TA and ST to generate STT can be determined by analyzing the peak changes of the material. The size and potential of STT, ST and tFNA were obtained using DLS. The morphologies of the materials under TEM and AFM were used to further analyze the size and shape differences between STT and tFNA.

### Stability of STs and STTs

The stability of ST and STT was demonstrated mainly by their serum stability and enzyme stability. First, special treatment of the materials was needed. siRNA and the synthesized ST and STT were mixed with FBS (0, 1, 2, 4, 6, 8 and 10%; v/v) at 37°C for 24 h. In addition, siRNA, ST and STT were incubated with FBS (10%, v/v) at 37°C for 0, 1, 3, 6, 12 and 24 h. Enzyme stability was achieved by mixing siRNA, ST and STT with RNase A (Sangon, Shanghai, China; 0 0.1, 0.5, 1, 2, 4, 8 and 16 U/mL) followed by incubation at 37°C for 24 h. siRNA, ST and STT were also incubated with 16 U/mL RNase A at 37°C for 0, 1, 3, 6, 12 and 24 h. Finally, all specially treated siRNA, ST and STT were subjected to AGE, and the bands were analyzed for changes in fluorescence and grayscale values using ImageJ.

In addition, considering that STT is ultimately applied to the surface of the skin for transdermal treatment, it is necessary to confirm the storage stability of STT. A 1000 nM STT solution was stored at room temperature and 4°C for 1 to 7 days. The final results were also demonstrated by AGE.’

### Uptake of Cy5 Loaded siRNAs, tFNAs, STs, and STTs

DCs and HaCaTs were grown in corresponding 12‐well plates with climbing plates and 6‐well plates, respectively. After adherent growth, 250 nM Cy5‐loaded siRNA, tFNA, ST, and STT were added to the medium. After 24 h of cultivation in the incubator, these cells were washed, and the DCs and HaCaTs in the 6‐well plate were digested and collected in corresponding flow cytometry tubes. Next, the entry efficiency of the four materials into two types of cells was measured using flow cytometry (FC500 Beckman, Illinois, USA). In addition, after washing, the cytoskeleton and nucleus were stained with phalloidin FITC for 30 min and DAPI for 10 min. Finally, the slides were placed under a confocal microscope (N‐SIM, Nikon, Tokyo, Japan) to obtain intracellular images.

### Escape of STTs from Lysosomes

After coincubation with 250 nM Cy5‐loaded siRNA, tFNA, ST, and STT for 24 hours, DCs and HaCaTs were washed. Then, the cells were further incubated in culture medium containing LysoTracker probes for 1 h. After cleaning, the colocalization of Cy5 and LysoTracker probes in living cells were observed by the confocal microscope.

### Transdermal Experiment in Mice

Cy5‐labeled siRNA, tFNA, ST, and STT were mixed with equal volumes of moisturizing cream (Vaseline, USA) and set aside. BALB/c mice (6‐8 weeks old, male) were subjected to shaving and hair removal after one day of cultivation. Next, the prepared cream was applied to the skin surface. The cream was replaced with a new cream every day and covered with a medical patch (3 M Tegaderm, USA). The skin tissue was removed on the second and seventh days and fixed in 4% paraformaldehyde for 24 h. Then, the skin tissue was frozen in liquid nitrogen, fixed with embedding glue, and cut into 5–10 µm tissue sections to be attached to slides. Afterward, at room temperature, the nucleus was stained on a glass slide with DAPI working solution for 10 min. The final slices were scanned using a scanner (Olympus Corporation, Japan). The animal experiment was approved by the Research Ethics Committee of West China Stomatological Hospital, Sichuan University.

### Immunofluorescence

DCs and HaCaTs were inoculated on cell slides and treated as mentioned earlier. The treated cells were successively fixed, permeabilized, and sealed with 4% paraformaldehyde (15 min) and 0.5% Triton X‐100 (20 min), and goat serum (1 h). Next, the cells were soaked in diluted antibodies against NF‐κB p‐p65 (1:100), TNF‐α (1:500), IL‐1β (1:50), NF‐κB p65 (1:100), and IL‐6 (1:500) and maintained overnight at 4 °C. The next day, the cells were stained with the secondary antibody (1:500, 1 h), FITC phalloidin (7:1000, 20 min) and DAPI (1:1000, 10 min) in the dark. Finally, the fluorescence images of related protein expression were obtained by a confocal laser microscope. In addition, all steps included washing three times with PBS.

### WB

The total protein of DCs and HaCaTs was extracted, and boiled to obtain the protein sample. Next, the protein samples were separated and transferred. The membrane containing protein expression was kept in a rapid blocking solution for 15 min and then transferred to the first antibody overnight. The next day, after rewarming for 30 min and the bands were then soaked to a second antibody dilution solution (Beyotime, Shanghai, China) for 1 hour. Finally, the band displayed the corresponding image in the imager under the exposure solution.

### Enzyme‐Linked Immunosorbent Assay (ELISA)

The collected cell supernatant and mouse serum samples were placed at −80 °C for storage. After washing, prepared samples and standard samples were added to the enzyme‐linked plate, and the samples were diluted according to the preexperiment. The subsequent operations were strictly performed according to the requirements of the ELISA detection kit (MULTI SCIENCES, Hangzhou, China). Finally, with the help of the developer and terminator, the pore plate exhibits a concentration‐dependent color change. The final result was obtained using a microplate reader (Thermo Fisher, MA, USA).

### Establishment and Treatment of Psoriasis

The back skin (approximately 2 cm*2 cm) of 5 BALB/c mice in each group was preshaved and depilated. After one day of recovery, the skin of the mice was treated with 80 mg IMQ cream (IMQ: 1 mg/cm^2^) every day for approximately 6 h. After the skin was cleaned, a cream containing therapeutic drugs was applied. After the operation was repeated for 6 days, the serum and skin tissue of the mice were taken for testing on the seventh day. The spleen tissue was extracted for flow cytometry analysis. Moreover, the PASI was recorded daily for each mouse according to Table S2, Supporting information.

### Statistical Analysis

One‐way ANOVA and t tests were used to analyze the statistical significance (p < 0.05) of the data in GraphPad Prism 6.01 (GraphPad Software Inc., San Diego, CA, USA).

## Conflict of Interest

The authors declare no conflict of interest.

## Supporting information



Supporting Information

## Data Availability

The data that support the findings of this study are available from the corresponding author upon reasonable request.
