# Supplementary material for: Transcutaneous Immunotherapy for RNAi: A Cascade‐Responsive Decomposable Nanocomplex Based on Polyphenol‐Mediated Framework Nucleic Acid in Psoriasis
Source: Adv Sci (Weinh). 2023 Oct 5;10(33):2303706. doi: 10.1002/advs.202303706 (PMC10667853; doi:10.1002/advs.202303706)
Supplement: Supplementary file 1 — Supporting Information [file ADVS-10-2303706-s001.pdf]

ADVANCED SCIENCE
Open Access

Supporting Information

for *Adv. Sci.*, DOI 10.1002/advs.202303706

Transcutaneous Immunotherapy for RNAi: A Cascade-Responsive Decomposable Nanocomplex Based on Polyphenol-Mediated Framework Nucleic Acid in Psoriasis

*Mei Zhang, Xin Qin, Yang Gao, Jiale Liang, Dexuan Xiao, Xiaolin Zhang, Mi Zhou and Yunfeng Lin**

Supporting Information

Transcutaneous Immunotherapy for RNAi: A Cascade-Responsive Decomposable Nanocomplex Based on Polyphenol-Mediated Framework Nucleic Acid in Psoriasis

*Mei Zhang[1], Xin Qin[1], Yang Gao[1], Jiale Liang[1], Dexuan Xiao[1], Xiaolin Zhang[1], Mi Zhou[1], and Yunfeng Lin[1]**

[1] State Key Laboratory of Oral Diseases, National Clinical Research Center for Oral Diseases, West China Hospital of Stomatology, Sichuan University, Chengdu 610041, P. R. CHINA.

Author list:

Mei Zhang. PhD. First author.

State Key Laboratory of Oral Diseases, West China Hospital of Stomatology, Sichuan University, Chengdu 610041, P. R. CHINA.

E-mail address: 1046134469@qq.com

Xin Qin. PhD. Author 2

State Key Laboratory of Oral Diseases, West China Hospital of Stomatology, Sichuan University, Chengdu 610041, P. R. CHINA.

E-mail address: 759362983@qq.com

Yang Gao. PhD. Author 3

State Key Laboratory of Oral Diseases, West China Hospital of Stomatology, Sichuan University, Chengdu 610041, P. R. CHINA.

E-mail address: 945162498@qq.com

Jiale Liang1. BD. Author 4

State Key Laboratory of Oral Diseases, West China Hospital of Stomatology, Sichuan University, Chengdu 610041, P. R. CHINA.

E-mail address: 1326932436@qq.com

Dexuan Xiao. PhD. Author 5
State Key Laboratory of Oral Diseases, West China Hospital of Stomatology, Sichuan University, Chengdu 610041, P. R. CHINA.
E-mail address: xdxuan1994@qq.com

Xiaolin Zhang PhD. Author 6
Department of Orthopedics, Orthopedic Research Institute, West China Hospital, Sichuan University, Chengdu 610041, P. R. CHINA.
E-mail address: 1522179366@qq.com

Mi Zhou. PhD. Author 7
Department of Orthopedics, Orthopedic Research Institute, West China Hospital, Sichuan University, Chengdu 610041, P. R. CHINA.
E-mail address: 820101715@qq.com

Yunfeng Lin. Prof. Corresponding Author*
State Key Laboratory of Oral Diseases, West China Hospital of Stomatology, Sichuan University, Chengdu 610041, P. R. CHINA.
College of Biomedical Engineering, Sichuan University, Chengdu 610041, P. R. China
E-mail address: yunfenglin@scu.edu.cn

Experimental Section

1.1. Materials.

The DC and HaCaT were obtained by ATCC. The single-strand DNAs (ssDNAs), siRNA, RNase H, RNase A and primer sequences designed for quantitative PCR were purchased from and purified by Sangon Biotech (Shanghai, China). TA and TNF-α were purchased from MCE (Shanghai, China). The lipopolysaccharides (LPS) was purchased from Sigma (Missouri, USA). In this experiment, all antibodies applied to the quantitative analysis of protein were purchased from Abcam (Cambridge, U.K.) and Cell Signaling Technology (Boston, USA). ELISA kits were obtained from MULTI SCIENCES (Hangzhou, China). The antibody MHC-II-PE,

CD11c-FITC, CD86-PE-Cy7, CD45-APC-Cy7, and CD80-APC were purchased from BD Biosciences (San Diego, CA, USA).

1.2. Isolation of BMDCs

After cervical dislocation, c57 mice (6-8 weeks old, male) were euthanized and soaked in 75% alcohol for 5 min. After fully exposing and isolating the leg bones, the cells in the bone marrow cavity were washed out with cold PBS by centrifuging for 5 min at 1500 rpm. Next, the cells were incubated with diluted red blood cell lysate and then washed with PBS. The final collected cells were resuspended in RPMI 1640 culture medium containing 20 ng/mL IL-4, GM-CSF and 10% FBS. We changed the medium on the 3rd and 5th days. On the seventh day, the cells suspended in the culture dish were removed for storage and were considered BMDCs.

1.3. Cell Culture

The DCs and HaCaTs, were cultured in RPMI 1640 medium containing 10% FBS and 1% penicillin-streptomycin. In addition, BMDCs extracted from mice were cultured in RPMI 1640 medium containing 20 ng/mL GM-CSF, IL-4 and 10% FBS. All cells were cultured in the incubator containing 5% CO^2 and 95% air at 37 °C. Next, an in vitro model of psoriasis was established using HaCaTs. According to the experimental requirements, we divided the cells into the following seven groups for the experiment: control group: no special treatment; TNF-α group: HaCaTs were incubated in medium supplemented with 20 ng/mL TNF-α for 36 h; Other drug groups: HaCaTs were cultured in the medium containing 20 ng/mL TNF-α for 12 h and then continued for 24 h in medium supplemented with additional drugs (250 nM tFNAs, 1000 nM siRNAs, 250 nM STs, 12.5 μg/mL TA, STTs (250 nM STs, 12.5 μg/mL TA)).

In addition, it is necessary to establish an inflammation model using DCs to verify the anti-maturation effect of STTs on DCs. According to the experimental requirements, we divided the cells into the following seven groups for the experiment: control group: no special treatment; LPS group: DCs were cultured in a medium containing 100 ng/mL LPS; Other drug groups: DCs were precultured in the medium containing drugs (250 nM tFNAs, 1000 nM siRNAs, 250 nM STs, 12.5μg/mL TA, STTs (250 nM STs, 12.5 μg/mL TA)) for 4 h, on this basis, DCs were continued to be cultivated for 24 h in the medium with additional 100 ng/mL LPS.

1.4. CCK-8 Assay

The HaCaTs were treated in groups: HaCaT cell was cultured in the medium with or without 20 ng/mL TNF-α for 12 h and then continued for 24h in medium supplemented with additional drugs. Later, at 37 °C, the cells were cultured in 10% (v/v) CCK-8 solution. Finally, the absorbances were measured at 450 nm to determine the cell viability.

1.5. Migration Assay

Scratch experiments were used to investigate cell migration behavior. HaCaTs (2×10^5) were cultured in12 plates. After washing with PBS, a pipette tip was used to create a cross-shaped scratch in each well. The cells were rewashed three times and cultured with RPMI 1640 medium without FBS containing different drugs for 24 h. Images of the samples were taken after 0 h and 24 h of treatment.

1.6. Cell Apoptosis Assay

The KeyGEN's Annexin V-FITC Apoptosis Detection Kit (Jiangsu, China) was selected to detect apoptosis of the HaCaTs. Then, the outcomes were analyzed by flow cytometry. After drug and TNF-α treatment, the cells were cleaned, digested and harvested into the corresponding centrifugal tubes. After twice cleaning, the cells were then resuspended in binding buffer (500 μL/tube) at 4 °C. In the dark, dyestuffs were added in sequence. Approximately 10−15 min later, the samples were tested by flow cytometry.

1.7. Quantitative PCR (qPCR)

The cells treated as previous were harvested in cellular lysis buffer (TRIzol, Invitrogen, Carlsbad, CA, USA). According to the manufacturer's instructions (RNA pure total RNA extraction kit, RP1202, BioTeke, Jiangsu, China), the RNA was extracted and purified. RNA was reverse transcribed into the cDNA based on PrimeScript RT reagent Kit (Takara Bio, Shiga, Japan). Then, the cDNA was added to the Mix required by qPCR. The program set by qPCR was run to obtain the final RNA expression result. The primer sequences of TNFα, IL6, IL-1β, and NF-κB are provided in Table S3.

1.8. Cell Morphology Detection

In order to detect the mature state of DCs, we further analyzed the morphology of DCs through scanning electron microscopy (SEM). DCs were seeded into a 24 well plate with climbing plates and grouped according to the requirements mentioned earlier. After the cell culture is completed, the cells are fixed with cold 4% paraformaldehyde for 20 min. Then, the cell

samples were subjected to dehydration treatment, specifically, 25% ethanol treatment for 15 min, 50% ethanol treatment for 15 min, 75% ethanol treatment for 15 min, followed by 100% ethanol treatment for 15 min. The samples were further dried and sprayed with gold, and finally images were obtained through SEM.

1.9. Determination of the Maturation Status of DCs

Flow cytometry was used to analyze the maturation status of DCs in spleen tissue and BMDCs extracted in vitro. The obtained spleen was immediately ground and passed through a 70 μm cell sieve to obtain a single-cell suspension. BMDCs were collected after grouping and culturing as mentioned earlier to obtain the single cells to be determined. After washing with PBS and performing lysis of red blood cells, these cells were resuspended in flow cytometry buffer and stained with $1*10^6$ cells per tube. After being blocked by FCR, the cells were incubated with anti-mouse antibodies (MHC II-PE, CD11c-FITC, CD86-PE-Cy7, CD45-APC-Cy7, and CD80-APC) in the dark for 30 min at room temperature. Finally, DCs labeled with CD80 and CD86 were analyzed using flow cytometry (BD LSRFortessa, BD, USA).

1.10. Distribution of Cy5-labeled siRNA, tFNAs, ST, and STT In Vivo

To ensure that STT can enter and remain in the skin, adult BALB/c mice (6-8 weeks old, male) were selected. The skin of mice was subjected to hair removal and then coated with Cy5-labeled siRNAs, tFNAs, STs, and STTs for 24 h. The next day, we wiped the surface of the mouse skin clean to ensure that there were no residual drugs. Next, mice anesthetized with isoflurane were placed under an IVIS (Bio Real Quick View 3000, Austria) for fluorescence imaging of the skin.

1.11. Histological Staining

The obtained skin tissue was fixed in a 4% paraformaldehyde solution for 48 h, then dehydrated in a disposable tissue embedding box, soaked in wax, and embedded. Then the tissue wax blocks were cut wax into 5 μm sections. Then, after dewaxing and hydration, the slices were stained with hematoxylin solution and alcohol eosin staining solution for several minutes each. IHC staining, on the other hand, involves sealing the section after hydration, repairing the antigen, and then staining with primary and secondary antibodies and DAPI. Finally, the stained sections were dehydrated with pure alcohol and then made the slices transparent by xylene for microscopic observation.

Table S1. Base sequence of each ssDNA and siRNA.

DNA	Base sequence (5′ → 3′)
S1	ATTTATCACCCGCCATAGTAGACGTATCACCAGGCAGTTGAGA CGAACATTCCTAAGTCTGAA
S2	ACATGCGAGGGTCCAATACCGACGATTACAGCTTGCTACACGA TTCAGACTTAGGAATGTTCG
S3	ACTACTATGGCGGGTGATAAAACGTGTAGCAAGCTGTAATCGA CGGGAAGAGCATGCCCATCC
S4	ACGGTATTGGACCCTCGCATGACTCAACTGCCTGGTGATACGA GGATGGGCATGCTCTTCCCG
Cy5-S1	Cy5-ATTTATCACCCGCCATAGTAGACGTATCACCAGGC AGTTGAGACGAACATTCCTAAGTCTGAA
S1`	TTGACCTGTGAATTATTTATCACCCGCCATAGTAGACGTATCAC CAGGCAGTTGAGACGAACATTCCTAAGTCTGAA
S2`	TTGACCTGTGAATTACATGCGAGGGTCCAATACCGACGATTAC AGCTTGCTACACGATTCAGACTTAGGAATGTTCG
S3`	TTGACCTGTGAATTACTACTATGGCGGGTGATAAAACGTGTAG CAAGCTGTAATCGACGGGAAGAGCATGCCCATCC
S4`	TTGACCTGTGAATTACGGTATTGGACCCTCGCATGACTCAACTG CCTGGTGATACGAGGATGGGCATGCTCTTCCCG
siRNA1 (mouse)	sense：GCGACAAGGUGCAGAAAGAdTdT<br>antisense：UUCACAGGUCAAUCUUUCUGCACCUUGUCGCdTdT
siRNA2 (human)	sense：CCAUCAACUAUGAUGAGUU-dTdT<br>antisense：UUCACAGGUCAAAACUCAUCAUAGUUGAUGGdTdT
Cy5-siRNA1	sense：Cy5- GCGACAAGGUGCAGAAAGAdTdT<br>antisense：UUCACAGGUCAAUCUUUCUGCACCUUGUCGCdTdT

Cy5-siRNA2	sense：Cy5-CCAUCAACUAUGAUGAGUU-dTdT	
	antisense：UUCACAGGUCAAAACUCAUCAUAGUUGAUGGdTdT	

Table S2. Evaluation of the severity of psoriasis by clinical symptom score PASI

Feature		Score
**A.**	**Skin thickness**	
**1.**	Smooth skin without wrinkles	0
**2.**	Slight wrinkles appear on the skin at the edges of the area where the medication is applied	1
**3.**	The skin in the area where the medication is applied appears slightly wrinkled	2
**4.**	The skin wrinkles in the area where the medication is applied further deepen	3
**5.**	On the basis of a skin thickness score of 3, mice also experienced weight loss or poor condition	4
**B.**	**Skin scalling**	
**1.**	The skin surface is smooth and free of scales	0
**2.**	Mild scaling of the skin in the area where the medication is applied	1
**3.**	The skin in the area where the medication is applied is completely covered with scales	2
**4.**	The skin in the area where the medication is applied is completely covered with scales, and the scales are further deepened	3
**5.**	On the basis of a skin scalling score of 3, mice also experienced weight loss or poor condition	4
**C.**	**Skin erythema**	
**1.**	The skin surface is smooth and free of erythema	0
**2.**	Mild erythemas of the skin in the area where the medication is applied	1
**3.**	The skin in the area where the medication is applied is completely covered with erythemas	2

**4.**	The skin in the area where the medication is applied is completely covered with scales, and the erythemas are further deepened	3
**4.**	On the basis of a skin erythema score of 3, mice also experienced weight loss or poor condition	4
**Cumulative score: The sum of three scores**		**12**

Table S3. Sequence of primers for q-PCR.

**mRNA**	**primer pairs (5′ → 3′)**
GAPDH	Forward TCATGACCACAGTCCATGCCATCA
	Reverse CCCTGTTGCTGTAGCCAAATTCGT
TNF-α	Forward CCTGCCCCAATCCCTTTATT
	Reverse CCCTAAGCCCCCAATTCTCT
IL-1β	Forward ACAACAGGAAAGTCCAGGCTA
	Reverse TGGCAGAAAGGGAACAGAA
IL-6	Forward ACTCACCTCTTCAGAACGAATTG
	Reverse CCATCTTTGGAAGGTTCAGGTTG
NF-κB p65	Forward CTGCCGCCTGTCCTTTCTCATC
	Reverse ATGTCCTCTTTCTGCACCTTGTCAC

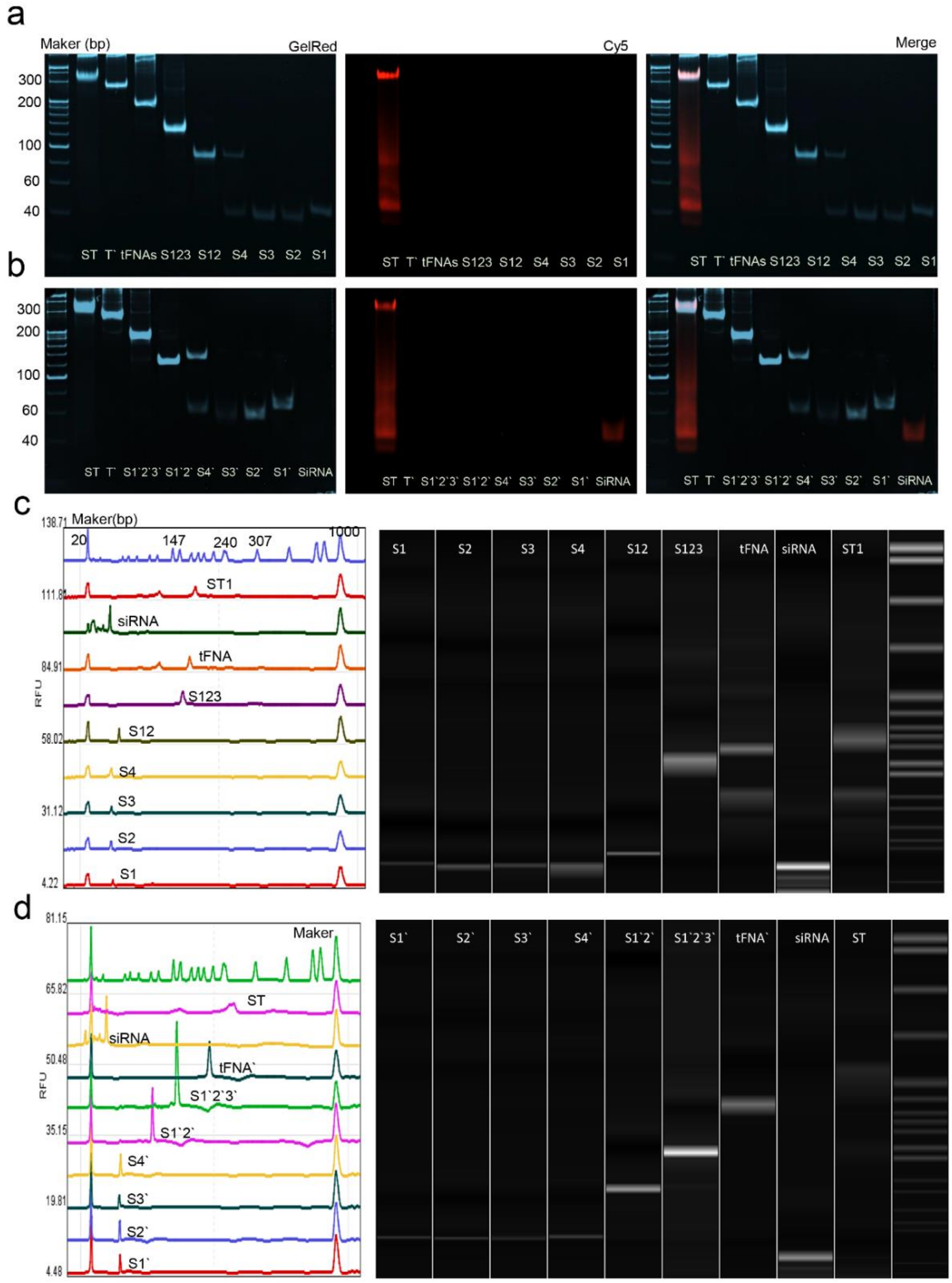

**Figure. S1 Synthesis of ST, tFNA and tFNA`.** (a) and (b) PAGE graph showing the successful synthesis of ST, tFNA and tFNA` (S12: S1+S2, S123: S1+S2+S3, T`: tFNA`, S1`2`: S1`+S2`, S1`2`3`: S1`+S2`+S3`). (c) and (d) CE graph showing the successful synthesis of ST, tFNA and tFNA` (S12: S1+S2, S123: S1+S2+S3, T`: tFNA`, S1`2`: S1`+S2`, S1`2`3`: S1`+S2`+S3`).

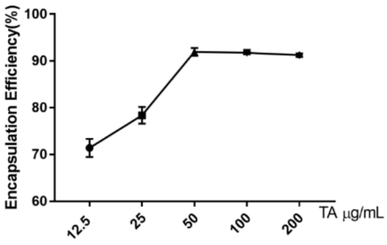

Figure. S2 The encapsulation efficiency of TA in STTs.

a

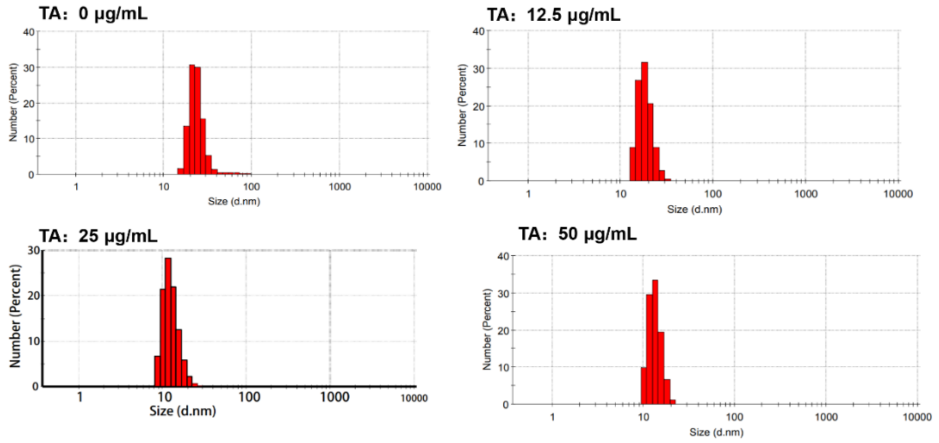

b

TA（μg/mL)	0	12.5	25	50
Diameter（nm)	27.40±4.86	22.34±4.32	18.77±5.70	15.20±2.33
PDI	0.378	0.566	0.520	0.340

c

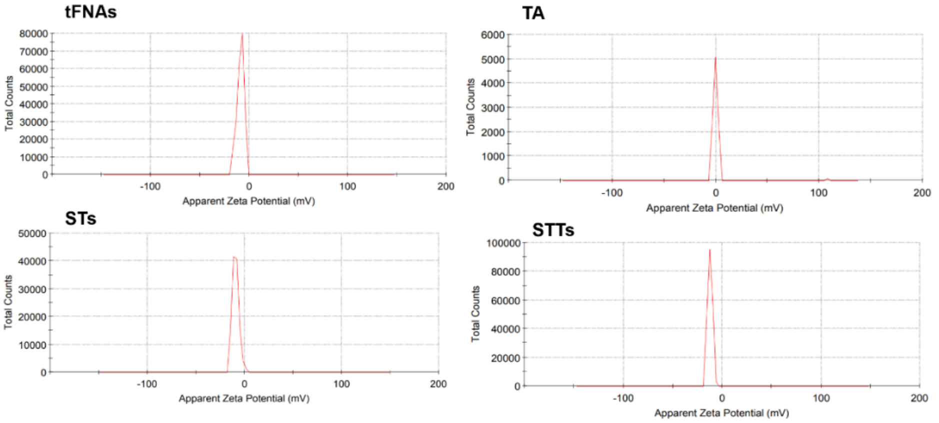

d

Material	tFNAs	TA	STs	STTs
Zeta potential（mV)	-8.94±3.58	-0.307±2.25	-9.34±3.43	-12.2±2.47

Figure. S3 The size and zeta potential of tFNAs and STTs. (a) and (b) Molecular size of STTs with different concentrations of TA measured by DLS. (c) and (d) Zeta potential of tFNAs, TA, STs and STTs measured by DLS.

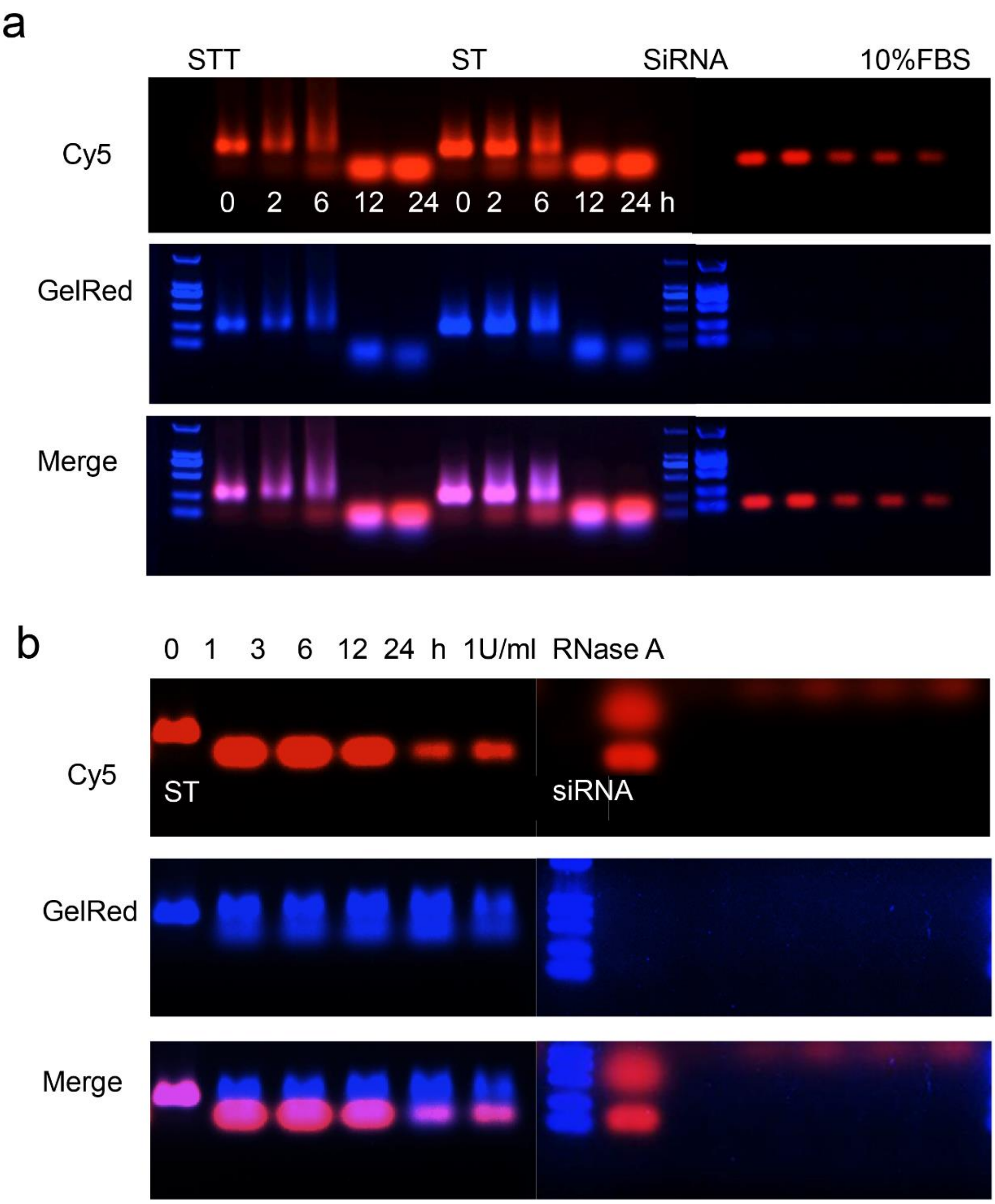

Figure. S4 The stability of siRNAs, STs and STTs. (a) Images of AGE showing Cy5 loaded free siRNAs, STs and STTs after incubation with 10% FBS for 0 h, 2 h, 6 h, 12 h, and 24h. (b) Images of AGE showing Cy5 loaded free siRNA and STs after incubation with 1 U/mL RNase A for 0 h, 1 h, 3 h, 6 h, 12 h, and 24h.

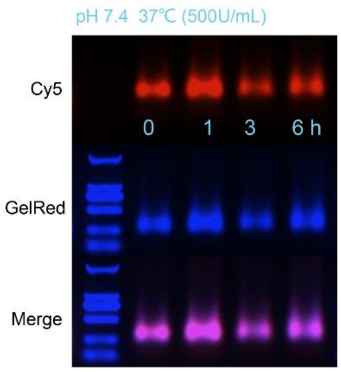

Figure. S5 Images of AGE showing the decomposition of Cy5-loaded STTs under 500 U/mL RNase H for 0, 1, 3, and 6 h at pH 7.4.

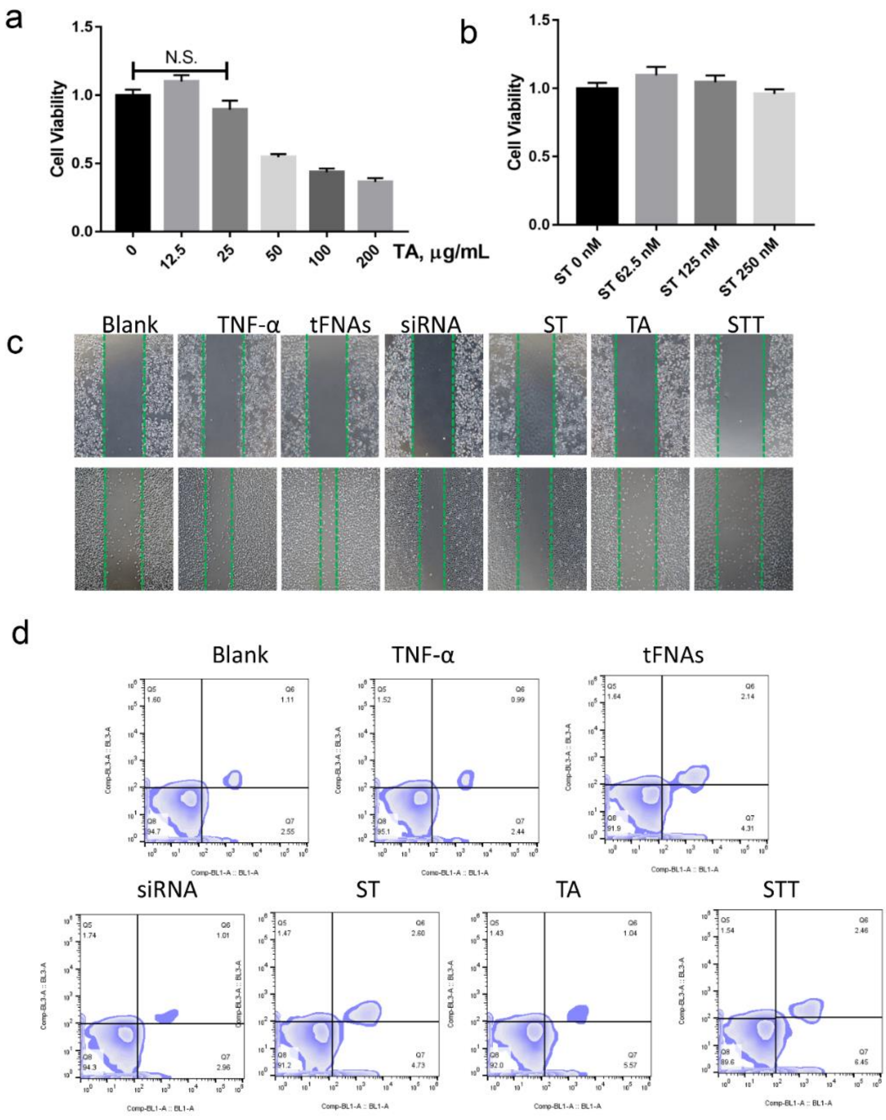

Figure. S6 The effect of STTs on the biological behavior of HaCaTs. (a) The statistical chart of CCK-8 showing the cell viability of HaCaTs under TA (0, 12.5, 25, 50, 100, 200 μg/mL).

(b) The statistical chart of CCK-8 showing the cell viability of HaCaTs under STs (0, 62.5, 125, 250 nM). (c) The scratch images showing the effect of STTs on the migration of HaCaTs. (d) Flow cytometry analysis showing the cell apoptosis of HaCaTs under different treatments.

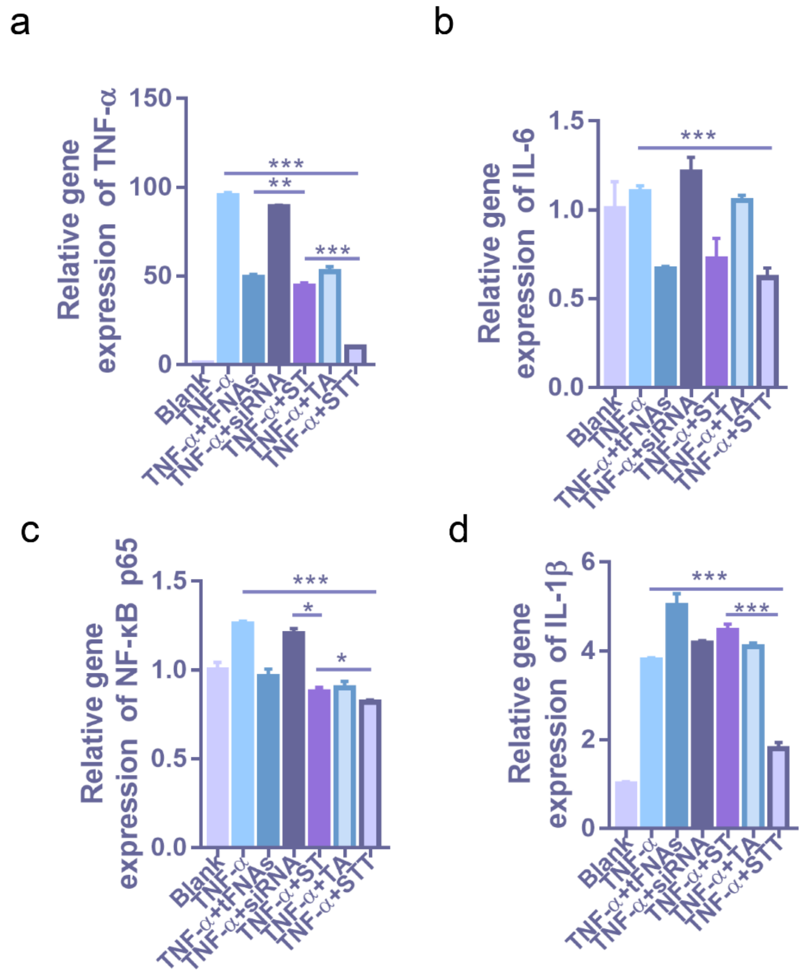

Figure. S7 (a)-(d) q-PCR analysis of the NF-κB p65, TNF-α, IL-6, and IL-1β expression level (n = 3)**.** Statistic differences are significant between the two groups ($p < 0.05$). Statistical analysis: *$P < 0.05$, **$P < 0.01$, ***$P < 0.001$.

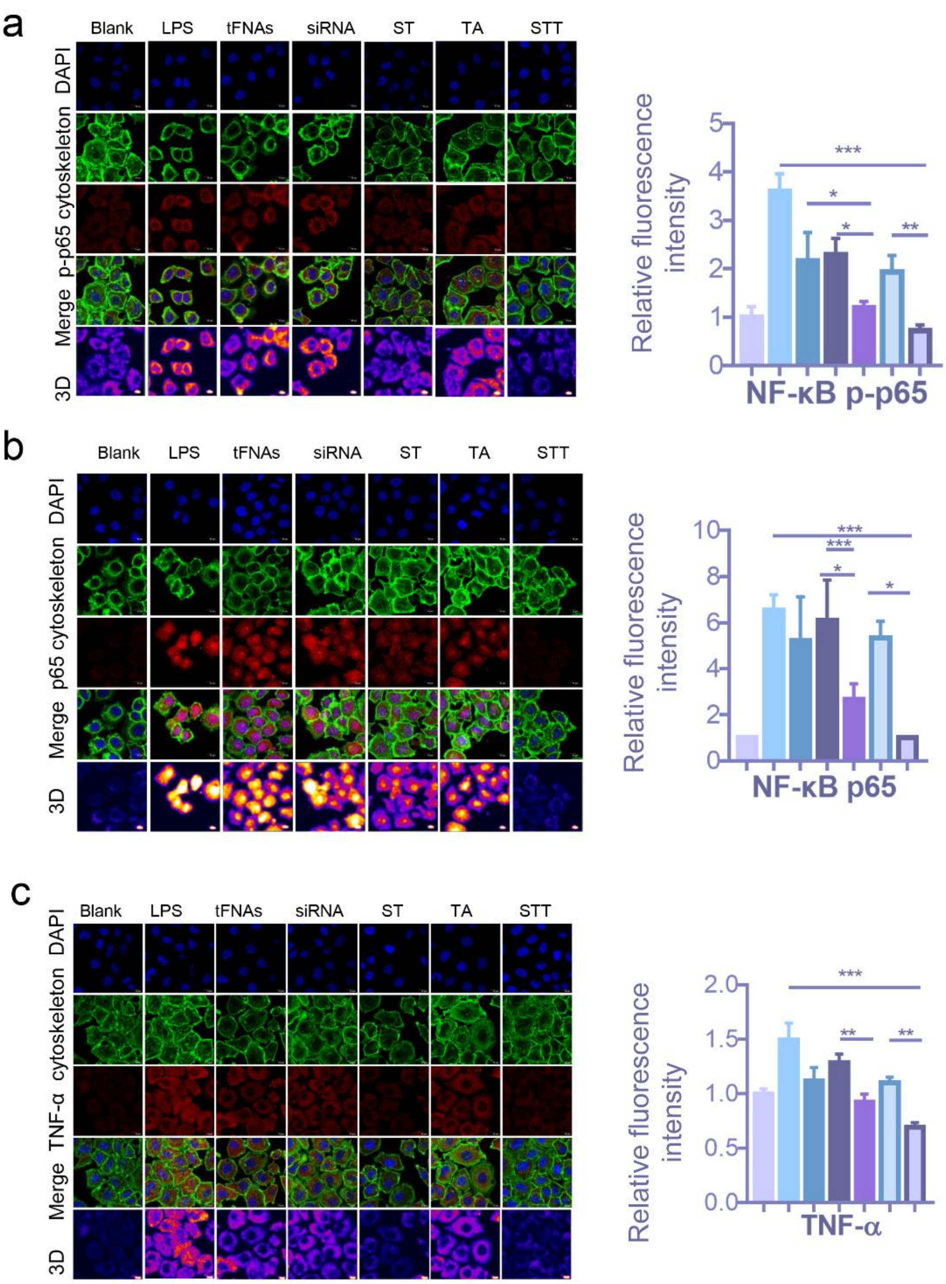

Figure. S8 (a), (b), and (c) Immunofluorescence images and its quantitative analysis of NF-κB p65, TNF-α, and NF-κB p-p65 in HaCaTs after different treatment for 24 h. (cytoskeleton: green; nucleus: blue; NF-κB p65, TNF-α, and NF-κB p-p65: red; 3D thermal imaging: 3D reconstruction of fluorescence microscopic images based on fluorescence intensity of ; NF-κB p65, TNF-α, and NF-κB p-p65). Scale bars are 10 μm.

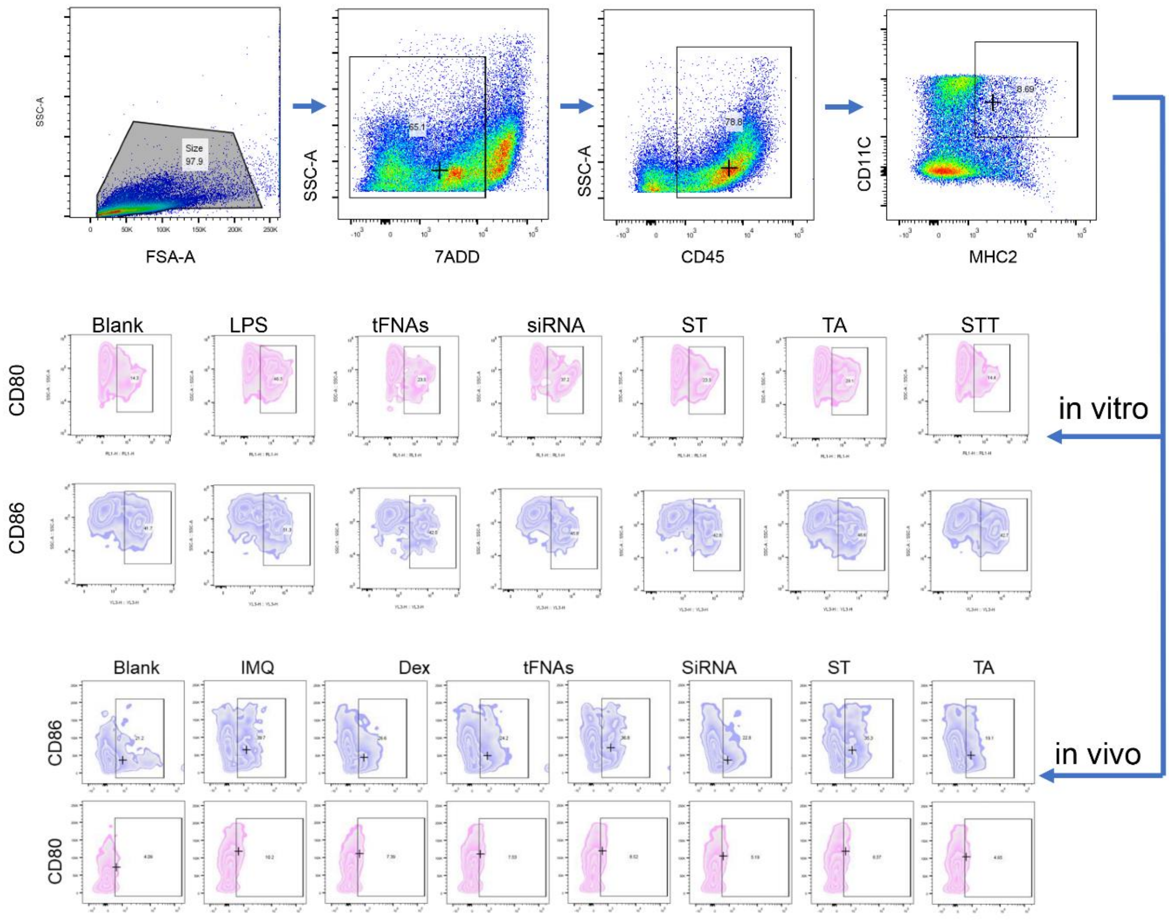

Figure. S9 Circle gate strategy of flow cytometry showing the changes in the DCs labeled with CD80 and CD86.

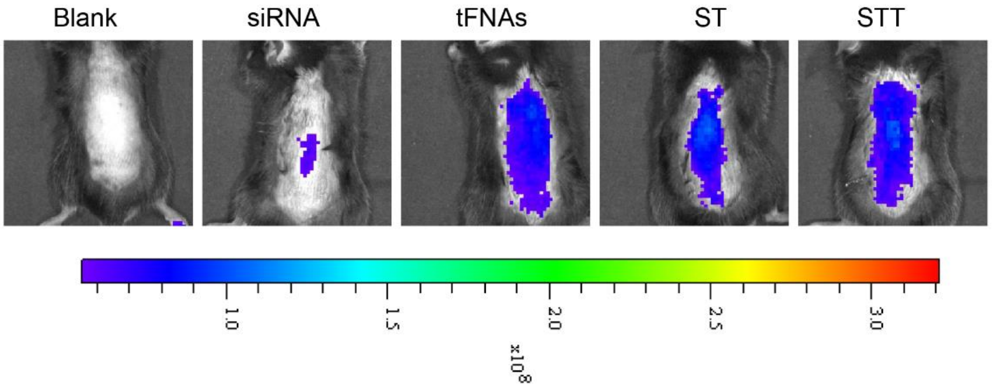

Figure. S10 Photographs of luminescence of skin after skin transdermal of Cy5-labeled siRNAs, tFNAs, STs and STTs.
